# Just a Fragment of Undescribed Diversity: Twenty New Oriental and Palearctic Species of Sciaroidea (Diptera), including DNA Sequence Data and Two New Fossil Genera

**DOI:** 10.3390/insects13010019

**Published:** 2021-12-23

**Authors:** Jan Ševčík, Heikki Hippa, Nikola Burdíková

**Affiliations:** 1 Faculty of Science, University of Ostrava, Chittussiho 10, CZ-710 00 Ostrava, Czech Republic; burdikova@seznam.cz; 2 Silesian Museum, Nádražní Okruh 31, CZ-746 01 Opava, Czech Republic; 3 Zoological Museum, Biodiversity Unit, University of Turku, FI-20014 Turku, Finland; heikki.hippa@gmail.com

**Keywords:** Diptera, Bibionomorpha, fungus gnats, taxonomy, DNA barcoding, oriental region, palearctic region

## Abstract

**Simple Summary:**

Two-winged flies (Diptera) are one of the megadiverse insect orders, in terms of biodiversity. The suborder Nematocera (=lower Diptera) includes several particularly species-rich groups, such as fungus gnats (Mycetophilidae and related families of the superfamily Sciaroidea) and gall midges (Cecidomyiidae), the latter presumably even being the most diverse family of Diptera of all. Although every year dozens of new species from these families are being described, either from extant fauna or from the fossil record, an amazing number of species still remain undescribed, especially outside Europe or North America. In this paper, another portion of new taxa of fungus gnats is described, enabling to better understand the fascinating and still little-known world of insects on Earth.

**Abstract:**

The following 17 extant new species of Sciaroidea (Diptera: Bibionomorpha) are described: *Bolitophila nikolae* Ševčík sp. nov. (Bolitophilidae, Taiwan), *Catocha jingfui* sp. nov. (Cecidomyiidae, Taiwan), *Catocha manmiaoe* sp. nov. (Cecidomyiidae, Taiwan), *Catocha shengfengi* sp. nov. (Cecidomyiidae, Taiwan), *Planetella taiwanensis* sp. nov. (Cecidomyiidae, Taiwan), *Diadocidia pseudospinusola* sp. nov. (Diadocidiidae, Taiwan), *Asioditomyia bruneicola* sp. nov. (Ditomyiidae, Brunei), *Asioditomyia lacii* sp. nov. (Ditomyiidae, Taiwan), *Ditomyia asiatica* sp. nov. (Ditomyiidae, Thailand), *Chetoneura davidi* sp. nov. (Keroplatidae, Brunei), *Euceroplatus mantici* sp. nov. (Keroplatidae, Thailand), *Setostylus fangshuoi* sp. nov. (Keroplatidae, Taiwan), *Platyceridion yunfui* sp. nov. (Keroplatidae, Hainan), *Terocelion adami* sp. nov. (Keroplatidae, Taiwan), *Hadroneura martini* sp. nov. (Mycetophilidae, Taiwan), *Paratinia furcata* sp. nov. (Mycetophilidae, Czech Republic, Slovakia), and *Nepaletricha sikorai* sp. nov. (Sciaroidea incertae sedis, Thailand). Two new genera are described from the mid-Cretaceous Burmese amber, *Burmasymmerus* gen. nov. (Ditomyiidae, type species *Burmasymmerus korneliae* sp. nov., including also *B. wieslawi* sp. nov.), representing the first record of the family Ditomyiidae from the Mesozoic, and *Burmatricha* gen. nov. (Sciaroidea incertae sedis, type species *Burmatricha mesozoica* sp. nov.). Molecular phylogeny of Ditomyiidae, based on two DNA markers (28S, COI), as well as that of *Catocha* Haliday, 1833, based on the mitochondrial COI and 16S fragments, are also presented.

## 1. Introduction

Fungus gnats (Diptera: Bibionomorpha: Sciaroidea) represent one of the most species-rich groups of Diptera [1]. The phylogenetic relationships among the families of Sciaroidea have been broadly discussed, and several hypotheses have been proposed (for a summary see Ševčík et al. [2]). Seven extant families are currently included in this superfamily: Bolitophilidae, Cecidomyiidae, Diadocidiidae, Ditomyiidae, Keroplatidae, Mycetophilidae, and Sciaridae. The former family Lygistorrhinidae was recently transferred to the family Keroplatidae as a subfamily [3].

Although considerable effort has been devoted to the study of the Oriental and Palearctic fungus gnats, large quantities of species still remain undescribed. If we take Taiwan as an example, we can roughly estimate that about 80% of species of Sciaroidea collected there represent undescribed species. Some taxa of Sciaroidea are better studied in the Oriental region as a whole, e.g., several genera of Mycetophilidae (e.g., References [4,5]), Keroplatidae [6,7], Sciaridae [8,9], and lower Cecidomyiidae [10], while the other taxa are still poorly known, especially those from the species-rich families Mycetophilidae, Keroplatidae, and Cecidomyiidae.

During the last twenty years, an extensive alcohol material of Oriental and Palearctic fungus gnats, as well as a relatively large collection of Burmese amber Diptera, has been accumulated by the first author. Only a small fraction of this amber collection has already been processed [11,12]. We, thus, take the opportunity to describe another portion of new species, from different families of Sciaroidea, including two new fossil genera from the mid-Cretaceous amber of Myanmar (=Burmese amber). DNA sequences are provided for all the new extant species described here. Phylogenetic trees based on DNA markers are also provided for several taxa of Cecidomyiidae and Ditomyiidae.

## 2. Materials and Methods

**Figure 1:**
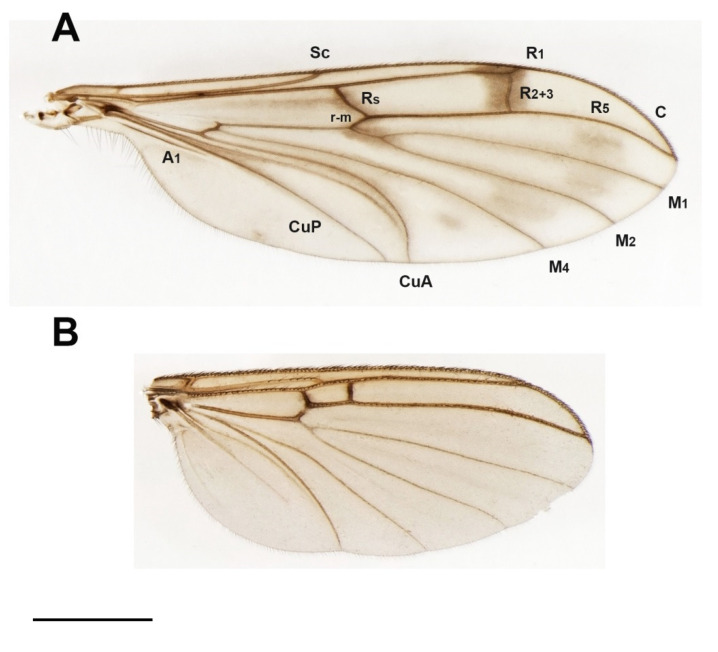
Wing photographs of two new extant species, with wing vein nomenclature indicated. (**A**). *Bolitophila nikolae* Ševčík sp. nov. (holotype); (**B**). *Hadroneura martini* sp. nov. (holotype, male). Abbreviations of veins: C—costal vein; Sc—subcostal vein; Rs—radial sector; R_1_—anterior branch of radius; R_2+3_—branches of radius; R_5_—third branch of radius; r-m—radio-medial vein; M_1_—first branch of media; M_2_—second branch of media; M_4_—fourth branch of media; CuA—anterior cubital vein; CuP—posterior cubital vein; A1—first branch of anal vein. Scale bar: 1 mm.

The morphological terminology principally follows that from the Manual of Afrotropical Diptera [13]. The abbreviations of wing veins are indicated in Figure 1.

The material is preserved in the following collections: Institute of Systematics and Evolution of Animals, Polish Academy of Sciences, Kraków, Poland (ISEA PAS); Jan Ševčík Lab, University of Ostrava, Ostrava, Czech Republic (JSL-OUC); Muséum National d’Histoire Naturelle, Paris, France (MNHN); National Museum, Prague, Czech Republic (NMPC); National Museum of Natural Science, Taichung, Taiwan (NMNS); Queen Sirikit Botanic Gardens, Chiang Mai, Thailand (QSBG); Silesian Museum, Opava, Czech Republic (SMOC); and Universiti Brunei Darussalam, Brunei (UBDC). The holotypes are mounted on microscopic slides in Euparal.

Molecular methods used in this paper follow those described in detail by Ševčík et al. [2,14]. A list of specimens used for DNA extraction and amplification is included in Table S1. The final dataset consists of 1219 characters (28S—561 bp, COI—658 bp) in the case of Ditomyiidae, and 1151 characters (16S—493 bp, COI—658 bp) in the case of Cecidomyiidae. The nuclear gene fragment was used in the case of Ditomyiidae, to reveal deeper relationships among particular genera. We selected as outgroup taxa two closely related genera to ingroup taxa, with more plesiomorphic character states.

This published work and the nomenclatural acts it contains have been registered in ZooBank, the online registration system for the ICZN. The LSID for this publication is: urn:lsid:zoobank.org:pub:20771259-D885-426D-A03E-60CD71711B9C.

## 3. Results and Discussion

### 3.1. Family Bolitophilidae

A small family of Sciaroidea, currently comprising some 60 extant species in a single genus *Bolitophila* Meigen, 1818, with two subgenera, *Bolitophila* s. str. and *Cliopisa* Enderlein, 1936 [15]. This is a principally Holarctic family, with three species recorded from Taiwan [16]. Here, we describe an additional Taiwanese species.

**Figure 2:**
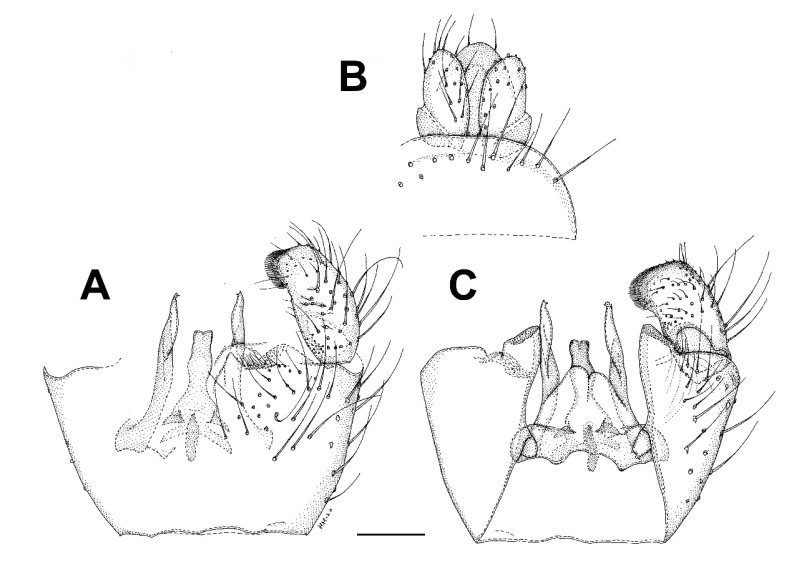
Male terminalia of *Bolitophila nikolae* Ševčík sp. nov. (holotype): (**A**) ventral view, (**B**) posterior part of abdomen in dorsal view, (**C**) dorsal view. Scale bar: 0.1 mm.

***Bolitophila* (*Bolitophila* s. str.) *nikolae*** Ševčík sp. nov. (Figure 1A and Figure 2).

LSID urn:lsid:zoobank.org:act:04A05F16-293F-4BD7-99AD-286276C3E55A.

**Type material.** Holotype: male, Taiwan (China), Nantou, Taroko National Park, Bilu, 2075 m, 4.v.–10.vi.2019, Malaise trap 3, S.-F. Lin, J. Ševčík & M. Tkoč leg. (NMNS, specimen after DNA extraction, mounted on slide in Euparal, No. JSBN20).

**DNA sequences.** DNA sequence (COI barcode region) taken from the holotype (specimen No. JSBN20) is deposited in GenBank. Its Accession number is provided in Table S1.

**Etymology.** This species is named after Ms. Nikola Burdíková (University of Ostrava, Czech Republic), a PhD student of the first author and co-author of this paper, for her achievements in the study of European Bolitophilidae and for her invaluable help with molecular methods used in this paper.

**Diagnosis.** This species can be easily recognized by the specific pattern of wing markings. It is the only currently known species of Bolitophilidae with markings on the wing membrane between veins M_1_, M_2_, and M_4_. The male terminalia are somewhat similar to those of *B. antennata* Ševčík & Papp, 2004, differing mainly in the shape of gonostylus.

**Description.** Male. Body mostly dark brown, wings with distinct dark markings (Figure 1). Wing length 5.3 mm. Head dark brown. Three ocelli in one line, median one smaller, distance between eye margin and lateral ocellus is about the diameter of the latter. Face twice as high as broad, brown, with setae on its lower margin. Mouthparts slightly prolonged. Maxillary palpus brownish, consists of a small palpifer and four longer palpomeres. Antenna long, almost as long as wing, with 14 flagellomeres. Flagellomeres all dark brown (F1 basally lighter), elongated, cylindrical, with dense setae about as long as the diameter of the flagellomere. F14 with a small apical appendage. Scape and pedicel brown, slightly shorter than wide. Scutum mostly brown, with a thin V-shaped light stripe. Distinct setae present along lateral margins and along V-shaped central stripe. Scutellum dark brown, with a subapical transverse row of setae. Mediotergite mostly brown, bare. Lateral sclerites dark brown and bare. Haltere dark brown, basally lighter, about as long as thorax. Wing (Figure 1A) with distinct markings. An extensive dark spot present around the apical part of R1 and on R_2+3_, also around Rs, r-m and the stem of M-fork, and between the branches of M. Less distinct dark areas present also in the proximal half of the wing, especially around the base of anal vein. Sc rather long, reaching to Rs. Vein C slightly produced beyond R5 to less than a quarter of the distance between the tips of R_5_ and M_1_. R_2+3_ ending perpendicularly in R_1_. A short medial crossvein (m-m) present. Basal part of M_4_ well developed. CuA downcurved towards the tip, A1 strong and straight, both reaching wing margin. Legs mostly dark brown, tibiae and tarsi lighter. Femora with longitudinal rows of dark setae. All tibiae with trichia arranged in dense longitudinal rows, without stronger setae. Tibial spurs formula 1.2:2. Abdomen mostly dark brown, tergites with pale thin apical bands. Terminalia (Figure 2) dark brown. Tergite 9 about twice as broad as long. Gonostylus distinctly shorter than gonocoxite, twice as long as wide, apically broadly rounded, with distinct apical comb of short dense dark setae. Parameres distinct and reaching beyond the posterior margin of gonocoxites. Aedeagal complex basally rather narrow, apically blunt, with a median furrow.

### 3.2. Family Cecidomyiidae

This is a huge family of Sciaroidea, currently comprising 6 subfamilies [17,18]. Species of the subfamily Cecidomyiinae, known for their gall-inducing larvae, have been widely studied worldwide, while the representatives of the lower gall midges, with mycophagous larvae, have been studied more intensively only during the last 20 years [10,19]. Here, we take the opportunity and describe three new Taiwanese species of *Catocha* Haliday, 1833, all of them occurring sympatrically in the same montane forest in Nantou province. A new species from the remarkable gall midge genus *Planetella* Westwood, 1840 (subfamily Cecidomyiinae, supertribe Cecidomyiidi), is also described based on a single male collected with a Malaise trap in Taiwan.

#### 3.2.1. Genus *Catocha* Haliday, 1833

A small genus of lower gall midges comprising 8 described species from the Holarctic and Oriental regions [17]. Three additional species are described below. Diagnostic characters of the genus are given, e.g., in Jaschhof and Jaschhof [19]. Phylogenetic relationships among the species of *Catocha* are discussed below (under *C. shengfengi* sp. nov.).

**Figure 3:**
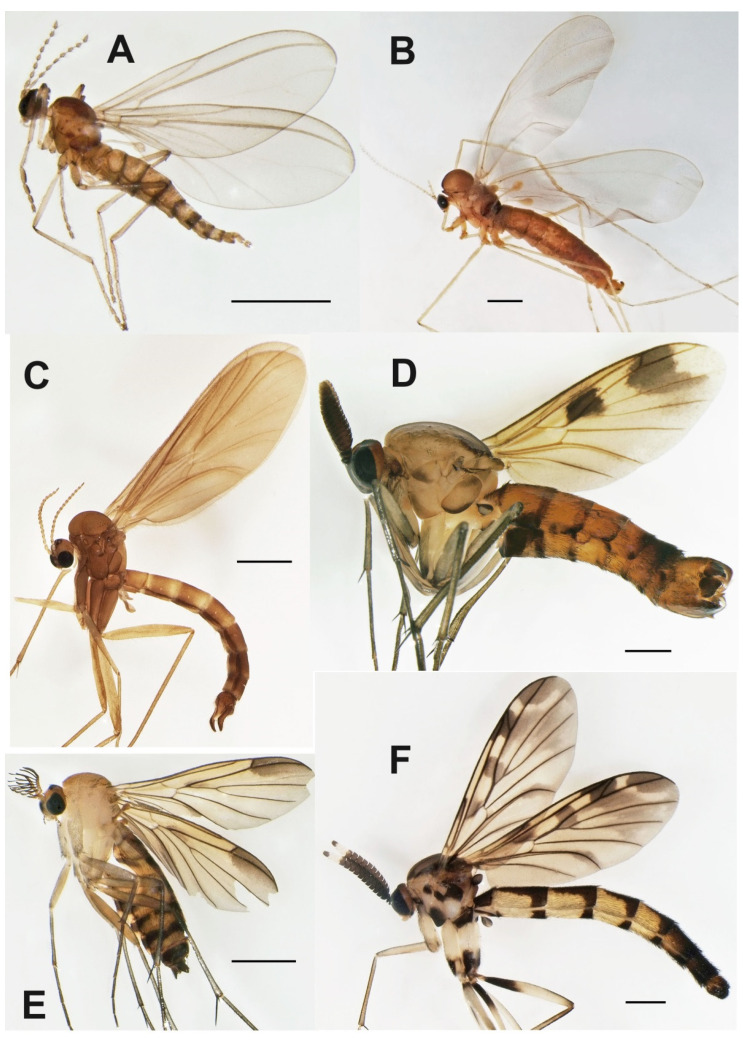
Habitus photographs of new extant species. (**A**) *Catocha jingfui* sp. nov. (paratype, female); (**B**) *Planetella taiwanensis* sp. nov. (holotype, male); (**C**) *Ditomyia asiatica* sp. nov. (paratype, male); (**D**) *Euceroplatus mantici* sp. nov. (holotype, male); (**E**) *Platyceridion yunfui* sp. nov. (paratype, female); (**F**) *Terocelion adami* sp. nov. (holotype, male). Scale bars: 1 mm. All photos by J. Ševčík.

**Figure 4:**
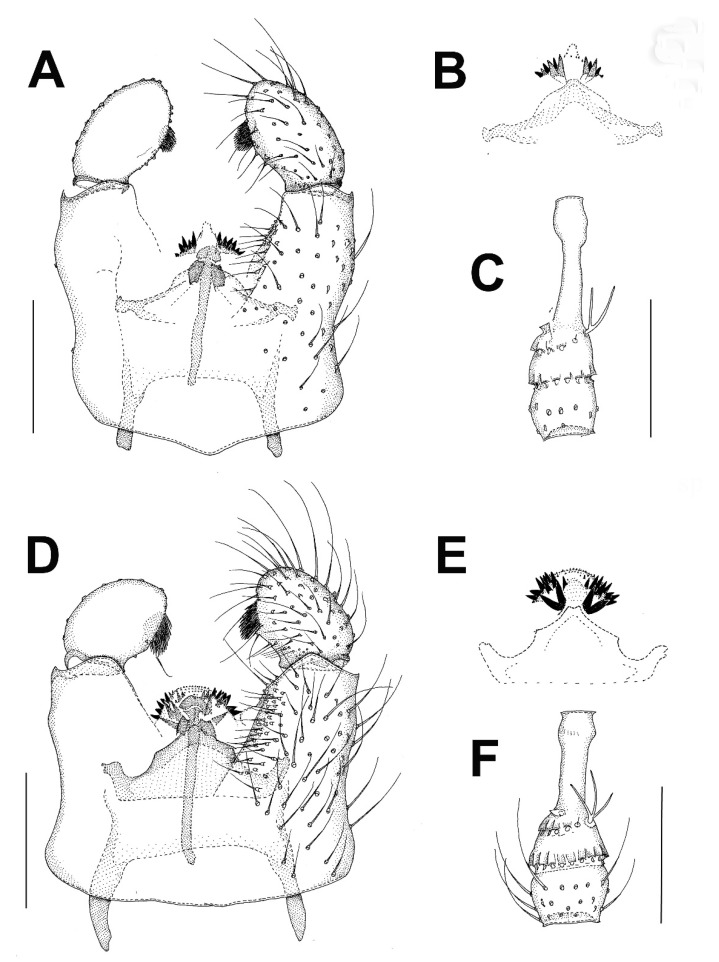
Male terminalia of new species of Cecidomyiidae. *Catocha jingfui* sp. nov. (holotype), (**A**) ventral view, (**B**) tegmen with associated structures in dorsal view, (**C**) fourth flagellomere; *Catocha manmiaoe* sp. nov. (holotype), (**D**) ventral view, (**E**) tegmen with associated structures in dorsal view, (**F**) fourth flagellomere. Scale bars: 0.1 mm.

***Catocha jingfui* sp. nov.** (Figure 3A and Figure 4A–C).

LSID urn:lsid:zoobank.org:act:4D7A9E78-4926-4327-B821-EEFAE92473E8.

**Type material.** Holotype: male, Taiwan (China), Nantou, Yuan Feng, 2700 m, 2–23.iv.2019, Malaise trap 1, S.-F. Lin, J. Ševčík & M. Tkoč leg. (NMNS, permanent preparation in Euparal, specimen after DNA extraction, No. JSTW32B). Paratype: female, the same data as holotype (specimen in ethanol after DNA extraction, No. JSTW32D, JSL-UOC).

**DNA sequences.** DNA sequences (COI and 16S mitochondrial genes) taken from the holotype (No. JSTW32B) and female paratype (JSTW32D) are deposited in GenBank. Their Accession numbers are provided in Table S1.

**Etymology.** This species is named after Dr. Jing-Fu Tsai, collection manager (Entomology) at the National Museum of Natural Science, Taichung. He hosted the first author in the museum and provided us with interesting material of Diptera collected in Taiwan.

**Diagnosis.** *Catocha jingfui* sp. nov. belongs to the *C. latipes* group as it is defined by Jaschhof and Jaschhof [19]. It is very similar to *C. shengfengi* sp. nov. but is morphologically distinguished by the aedeagal teeth which are only half of the size in the former and by having the neck of the antennal flagellomeres longer than the body, not vice versa.

**Description.** Male. Wing length 2.4 mm. Head capsule, thorax and terminalia brown, other parts, including wing, greyish brown. Head. Number of postocular long setae ca. 6, not much stronger than the other occipital setae. Clypeus with ca. 30 setae. Eye bridge complete, 3–4 facets wide, similar medially and laterally. 3 ocelli present, all well-developed. Maxillary palpus with 4 palpomeres, palpomere 1 with at least 2, palpomere 2 with no discernible hyalinous sensilla. Antennal flagellomere 4 with the neck longer than the body (Figure 4C), microtrichia present only on extreme base, setae on basal part of the body few and not arranged in whorls, medially on the body a complete crenulated whorl of ca. 13 setae, on distal part of the body with an incomplete crenulated whorl of ca. 7 setae, hyalinous sensilla on the apical part of the body three-branched (on more apical flagellomeres the hyalinous sensilla are bifurcate or even simple). Wings as in Figure 2A. Terminalia (Figure 4A,B). Gonocoxites in dorsal or ventral view constricted on anterior half, with a translucent transverse window-like part medio-ventrally on anterior half. Gonostylus ovoid, slightly longer than broad, the setosity normal, the subapical mesial tooth-like aggregation of trichia rather broad, far from the apex. Tegmen subtriangular, with angulate sides. Ejaculatory apodeme posteriorly flanked by dark sclerites. The aedeagal teeth medially very small, dark, laterally elongate triangular in shape, dark, about 3–4 times as long as basally broad, about 7 on each side, laterodorsally 2 conspicuously larger teeth on each side. Female. Similar to male in most respects. Antenna with 8 flagellomeres (Figure 3A).

***Catocha manmiaoe* sp. nov.** (Figure 4D–F).

LSID urn:lsid:zoobank.org:act:951BEE16-82DF-47CE-9907-5B353D231D26.

**Type material.** Holotype: male, Taiwan (China), Nantou, Yuan Feng, 2700 m, 23–28.iv.2019, Malaise trap 2, J. Ševčík & M. Tkoč leg. (NMNS, permanent preparation in Euparal, specimen after DNA extraction, No. JSTW32I). Paratype: male, the same data as holotype (SMOC, permanent preparation in Euparal, specimen after DNA extraction, No. JSTW32L).

**DNA sequences.** DNA sequences (COI and 16S mitochondrial genes) taken from the holotype and paratype are deposited in GenBank. Their Accession numbers are in Table S1.

**Etymology.** This species is named after Dr. Man-Miao Yang (National Chung Hsing University, Taichung) for her achievements in the study of Taiwanese gall midges.

**Discussion.** *Catocha manmiaoe* sp. nov. belongs to *C. latipes* group as it is defined by Jaschhof and Jaschhof [19]. The species is distinguished from the other known species of *C. latipes* group by the fan-like aggregation of unusually long lanceolate aedeagal teeth. By the lack of vein R_5_ it differs from all other *Catocha* except *C. brachycornis* (Spungis and Jaschhof). Except for the aedeagal teeth the two species differ by the length of the necks on the antennal flagellomeres which are ca. twice as long in *C. brachycornis* than in *C. manmiaoe* sp. nov.

**Description.** Male. Wing length 2.1 mm. Head capsule, thorax and terminalia brown, other parts, including wing, greyish brown. Head. The number of postocular long setae ca. 5. Clypeus with 15 setae. Eye bridge complete, 3–4 facets wide, similar medially and laterally. No ocelli observable but the specimen is broken on the vertex. Maxillary palpus with 4 palpomeres, palpomere 1 with three, palpomere 2 with one hyalinous sensillum. Antennal flagellomere 4 (Figure 4F) with the body longer than the neck, microtrichia present only on extreme base, setae on basal part of the body few and not arranged in whorls, medially on the body a complete crenulated whorl of ca. 12 setae, on distal part of the body with an incomplete crenulated whorl of ca. 5 setae, the hyalinous sensilla on the apical part of the body bifurcate (on more apical flagellomeres the hyalinous sensilla are at least mostly simple). Wing ratio length/width 2.4. Costal break present. M_2_ absent. Rs shorter than r-m, r-m weak net reaching R_1_. CuA2 evenly curved on distal part. Wing membrane sparsely setose. Terminalia (Figure 4D,E). Gonocoxites in dorsal or ventral view constricted on anterior half, without a translucent window-like part medio-ventrally on anterior half. Gonostylus ovoid, slightly longer than broad, the setosity normal, the subapical mesial tooth-like aggregation of trichia rather narrow, not far from the apex. Tegmen subtriangular with straight sides. Ejaculatory apodeme posteriorly not flanked by dark sclerites. The aedeagal teeth medially very small, dark, laterally lanceolate in shape, several times longer than broad, pale with dark apices, altogether ca. 12 in number and forming a fan-like aggregation. Female. Unknown.

**Figure 5:**
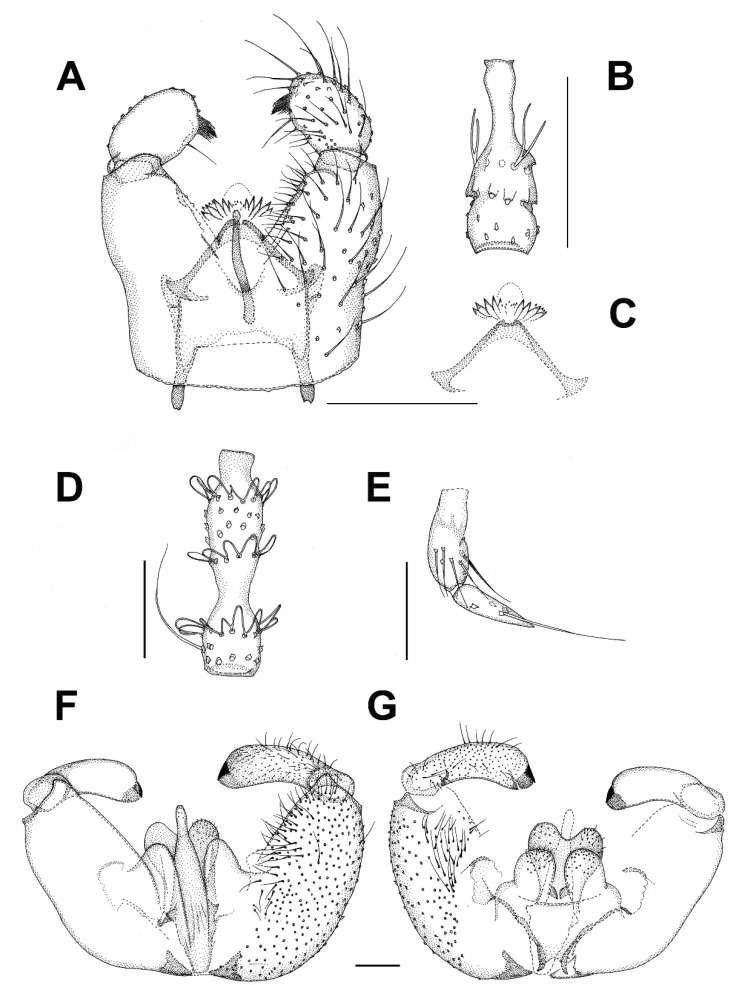
*Catocha shengfengi* sp. nov. (holotype): (**A**) ventral view, (**B**) fourth flagellomere, (**C**) tegmen with associated structures in dorsal view. *Planetella taiwanensis* sp. nov. (holotype): (**D**) fourth flagellomere, (**E**) maxillary palpus in lateral view, (**F**) male terminalia in ventral view, (**G**) male terminalia in dorsal view. Scale bars 0.1 mm.

***Catocha shengfengi* sp. nov.** (Figure 5A–C).

LSID urn:lsid:zoobank.org:act:44B4ABA6-8207-46DA-8339-BDBE53271444.

**Type material.** Holotype: male, Taiwan (China), Nantou, Yuan Feng, 2700 m, 2–23.iv.2019, Malaise trap 1, S.-F. Lin, J. Ševčík & M. Tkoč leg. (NMNS, permanent preparation in Euparal, specimen after DNA extraction, No. JSTW32). Paratype: male, the same data as holotype (SMOC, permanent preparation in Euparal, specimen after DNA extraction, No. JSTW32C).

**DNA sequences.** DNA sequences (COI and 16S mitochondrial genes) taken from the holotype (No. JSTW32) and paratype (JSTW32C) are deposited in GenBank. Their Accession numbers are in Table S1.

**Etymology.** This species is named after Dr. Sheng-Feng Lin (National Chung Hsing University, Taichung), who installed the Malaise trap into which type specimens of this species were collected. He also operated further Malaise traps in Taiwan and provided us with the material of Diptera from these traps.

**Diagnosis.** *Catocha shengfengi* sp. nov. belongs to the *C. latipes* group as it is defined by Jaschhof and Jaschhof [19]. It is very similar to *C. jingfui* sp. nov. For distinguishing characters, see under the latter. Both these species differ from *C. incisa* Jaschhof and Jaschhof by having the aedeagal teeth larger and angled lateral margin of tegmen, from *C. latipes* Haliday by simple, not angulate, lateral margin of tegmen and from *C. latipes* and *C. angulata* Jaschhof and Jaschhof and *C. subalpina* Jaschhof and Jaschhof by having all the aedeagal teeth directed obliquely posterior, not partly laterad and obliquely anteriad. It seems that *C. shengfengi* sp. nov. and *C. jingfui* sp. nov. differ from the other mentioned species by having a pair of strong sclerites flanking the posterior part of the ejaculatory apodeme but they may have been unobserved in the description; in the description of *C. subalpina* Figure 23F in Reference [19]) there are signs of these sclerites.

**Description.** Male. Wing length 2.6 mm. Head capsule, thorax abdominal tergites and terminalia brown, antenna, mouthparts, legs and abdominal sternites pale brown, wing pale yellowish brown. Head. Number of postocular long setae ca. 5. Clypeus with ca. 30 setae. Eye bridge complete, 3–4 facets wide, similar medially and laterally. 3 ocelli present, all well-developed. Maxillary palpus with 4 palpomeres, palpomere 1 with ca. 10, palpomere 2 with 2 hyalinous sensilla. Antennal flagellomere 4 with the body longer than the neck (Figure 5B), microtrichia present only on extreme base, setae on basal part of the body many and not arranged in whorls, medially on the body a complete crenulated whorl of ca. 16 setae (broken off in the mount), on distal part of the body with an incomplete crenulated whorl of ca. 7 setae, hyalinous sensilla on the apical part of the body three-branched (on more apical flagellomeres the hyalinous sensilla are bifurcate or even simple). Wing ratio length/width 2.4. Costal break present. M-fork present, one fourth of the total length of M. Rs shorter than r-m, r-m well-developed. CuA2 roundly angularly curved on distal part. Wing membrane setose, the setae relatively numerous. Terminalia (Figure 5A,C). Gonocoxites in dorsal or ventral view constricted on anterior half, with a weak translucent window-like part medio-ventrally on anterior half. Gonostylus ovoid, slightly longer than broad, the setosity normal, the subapical mesial tooth-like aggregation of trichia rather broad, far from the apex. Tegmen subtriangular with angulate sides. Ejaculatory apodeme posteriorly flanked by dark sclerites. The aedeagal teeth medially very small, many, dark, laterally elongate triangular in shape, dark, about 3–4 times as long as basally broad, about 10 on each side, laterodorsally 2 conspicuously larger teeth on each side. Female. Unknown.

**Figure 6:**
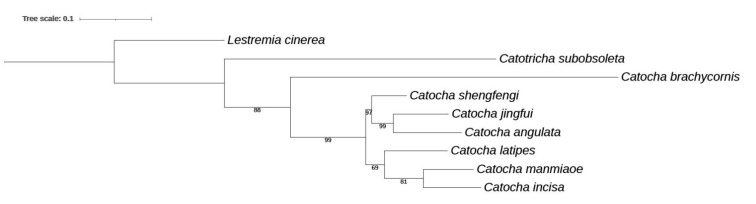
Phylogenetic relationships (Maximum Likelihood hypothesis) among the species *Catocha* based on two DNA markers (16S and COI).

**Phylogenetic relationships.** Phylogenetic relationships among the species *Catocha* are reconstructed in Figure 6, based on two mitochondrial DNA markers (COI and 16S). Some of the relationships are, however, insufficiently supported. The species of *Catocha* apparently underwent a rapid evolution, which may be difficult to reveal by these two mitochondrial markers. This tree, thus, should be considered only as preliminary, pending a more detailed molecular study of the lower gall midges as a whole.

#### 3.2.2. Genus *Planetella* Westwood, 1840

A relatively large Holarctic genus of remarkable gall midges, representing the largest gall midges in terms of adult body size (sometimes more than 1 cm), comprising 52 described species, none of them described after the year 1926 [17]. Almost all of them occur either in Europe or in North America, and they are in need of revision, focused on detailed structures of the male terminalia, in combination with DNA sequence data.

Only one species, tentatively placed in *Planetella* by Gagné and Jaschhof [17], was described from eastern Asia (Japan), originally as *Trishormomyia bambusae* Felt, 1932. The type series includes two male syntypes deposited in the National Museum of Natural History, Washington, DC, USA. According to Raymond Gagné (pers. comm.), they do not have a forward- projecting thorax, so that the prothorax is on the same vertical plane as the forelegs, as in the more usual Cecidomyiidi. It is, thus, not certain if *T. bambusae* even belongs to the genus *Planetella* but it definitely represents a different species, and most probably also a genus, than *P. taiwanensis* sp. nov., described below. While all the species of *Planetella*, where a host plant is reliably known, are associated with *Carex* spp., the host of the new species described here remains unknown.

***Planetella taiwanensis* sp. nov.** (Figure 3B and Figure 5D–G).

LSID urn:lsid:zoobank.org:act:14532D64-85A2-4E21-9129-2866390FC300.

**Type material.** Holotype: male, Taiwan (China), Nantou, Taitung, Tianlong trail, broad-leaved evergreen secondary forest, 920 m, 5.v.–8.vi.2016, Malaise trap, W. T. Yang leg. (NMNS, permanent preparation in Euparal, specimen after DNA extraction, No. JSPLA5).

**DNA sequence.** DNA sequence (COI barcode region) taken from the holotype (No. JSPLA5) is deposited in GenBank. Its Accession number is provided in Table S1.

**Etymology.** The specific name refers to Taiwan, where the holotype was collected.

**Diagnosis.** This species represents a typical species of *Planetella*, characterized by its large body size, ochreous coloration, and scutum produced above the head. It differs from all the other species in details on the male terminalia (especially the shape of cerci, hypoproct, and the apex of gonostylus), in combination with unique COI sequence.

**Description.** Male. Wing length 7.2 mm. Head. Brown, antenna pale yellowish brown, scape and pedicel little darker than flagellum, maxillary palpus pale yellowish brown. Eye bridge lacking. Antennal flagellum with 12 binodal tricircumfilial flagellomeres, apically with short uninodal flagellomere lacking circumfila. Flagellomere 4, Figure 5D loops of circumfila subequal, ca. 10 in number. Clypeus with a few ventral setae. Labrum with ca. 40 conspicuous, long, curved setae. Maxillary palpus (Figure 5E with 2 almost equally long palpomeres, basal palpomere with ca. 10, apical palpomere with 4 setae. Thorax and legs in bad condition, greatly faded, partly collapsed and details impossible to describe; it seems that there is a dark medial line anteriorly on mesonotum, that the posterior part of pleura and all of metanotum is very dark, and trochanters are dark. Wing. Pale yellowish brown. Costal break obscure. R and R_1_ subequal in length. M indistinct. CuA very weak, not forked. Halter pale, yellowish. Abdomen. Pale yellowish brown. Terminalia, Figure 5F,G. Gonocoxite very pale brown, gonostylus brown with darker apical part, all medial part of terminalia brown. Gonocoxites richly setose dorsally and ventrally, with rounded nonsetose lobe anteromedially, gonocoxal apodemes medially fused, with a subtriangular very dark sclerotization anteromedially. Gonostylus curved, evenly broad throughout, setosity similar to gonocoxa even if shorter, apical tooth subtriangular, black. Cerci short, rounded, with very short setae. Hypoproct heart-shaped, setae similar to cercus. Aedeagus very elongate subtriangular, apodemes strongly sclerotized, very dark, aedeagal teeth very small, subapical. Female. Unknown.

### 3.3. Family Diadocidiidae

This is a small family of Sciaroidea of uncertain phylogenetic position, currently comprising a single extant genus, with three subgenera [20]. Oriental species of Diadocidiidae were treated by Papp and Ševčík [21]. An additional species is described below.

**Figure 7:**
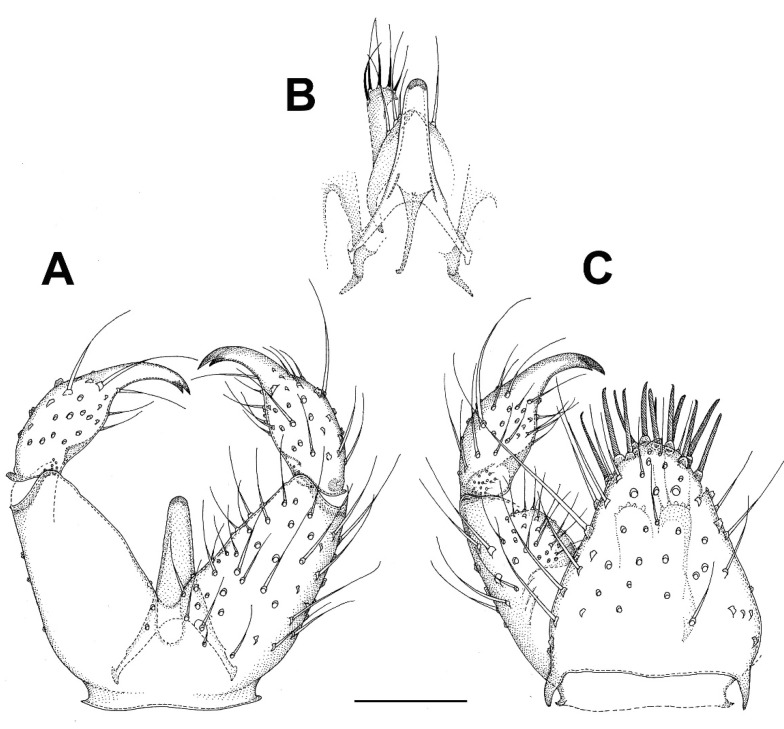
Male terminalia of *Diadocidia pseudospinusola* sp. nov. (holotype): (**A**) ventral view, (**B**) aedeagus, hypoproct and cerci in ventral view, (**C**) dorsal view.. Scale bar 0.1 mm.

***Diadocidia pseudospinusola* sp. nov.** (Figure 7).

LSID urn:lsid:zoobank.org:act:CD0E2C5F-B203-4A8A-98AE-3C0901314C6D.

**Type material.** Holotype: male, Taiwan (China), Nantou, Yuan Feng, 13.v.2018, J. Ševčík leg., sweeping vegetation (specimen after DNA extraction, No. JSTW28B, mounted on slide in Euparal), coll. NMNS. Paratype: male, Taiwan (China): Nantou: Taroko National Park, 26.iv.2019, J. Ševčík leg., sweeping vegetation (specimen in ethanol after DNA extraction, No. JSTW28, JSL-OUC).

**DNA sequences.** DNA sequences (COI barcode region) taken from the holotype (No. JSTW28B) and paratype (JSTW28) are deposited in GenBank. Their Accession numbers are provided in Table S1.

**Etymology.** The name of the new species refers to the similarity with *D. spinosula*.

**Diagnosis.** This species belongs to the subgenus *Diadocidia* sensu stricto and it is morphologically almost indistinguishable from the Palaearctic *Diadocidia spinosula* Tollet, 1948, sharing the same shape of tergite 9, including the strong posterior setae, and overall structure of the male terminalia. Both species differ in the shape of aedeagus, which is slightly narrower in *D. pseudospinosula* sp. nov., and in the structure of the apical part of the gonostylus (dorsal branch of the apical fork is shorter in the new species).

**Description.** Male. Wing length 4.1 mm (holotype). Head dark brown. Three ocelli, closely set on a subtriangular tubercle. Lateral ocellus larger than median one, separated from eye margin for distance about 0.8 times its diameter. Antenna with 14 flagellomeres, F1 about three times as long as broad, basally yellowish. The rest of flagellomeres dark brown, about twice as long as broad. Scape and pedicel yellowish brown, about as long as wide. Maxillary palpus with four palpomeres, their relative lengths are 1:1:2:3. Thorax all dark brown. Scutum weakly arched, setose. Scutellum with a row of subapical bristles. Mediotergite and all lateral sclerites bare. Haltere dark. Wing without markings, its membrane covered with macrotrichia. Wing venation the same as in other *Diadocidia* s. str. Legs mostly yellowish brown, except for darker mid and hind coxae. Femora and tibiae yellowish brown, irregularly covered with trichia. Tibial spurs (1:2:2) dark brown. Abdomen all dark brown. Terminalia (Figure 7) dark brown. Tergite 9 subtriangular, slightly longer than broad. Gonocoxites basally fused. Gonostylus almost as long as gonocoxite, tapering, with a distinct apical fork, with its dorsal branch shorter that the ventral one.

### 3.4. Family Ditomyiidae

This is a small and ancient family of Sciaroidea, currently comprising almost 100 extant species in 8 genera. Asian species of Ditomyiidae were studied in detail by Saigusa [22,23]. Additional taxa, both extant and fossil, are described below. A preliminary molecular phylogeny of the family is also presented here.

#### 3.4.1. Genus *Asioditomyia* Saigusa, 1973

An exclusively eastern Asian genus of Ditomyiidae, with only a single species hitherto described [22]. Two additional species are described below, indicating that it is a widespread genus in South-East Asia. *Asioditomyia* differs from the other genera of Ditomyiidae mainly in the short palpi, with only one palpomere, in the absence of both crossvein r-m and r-m fusion, and in the slightly compressed flagellomeres [22].

**Figure 8:**
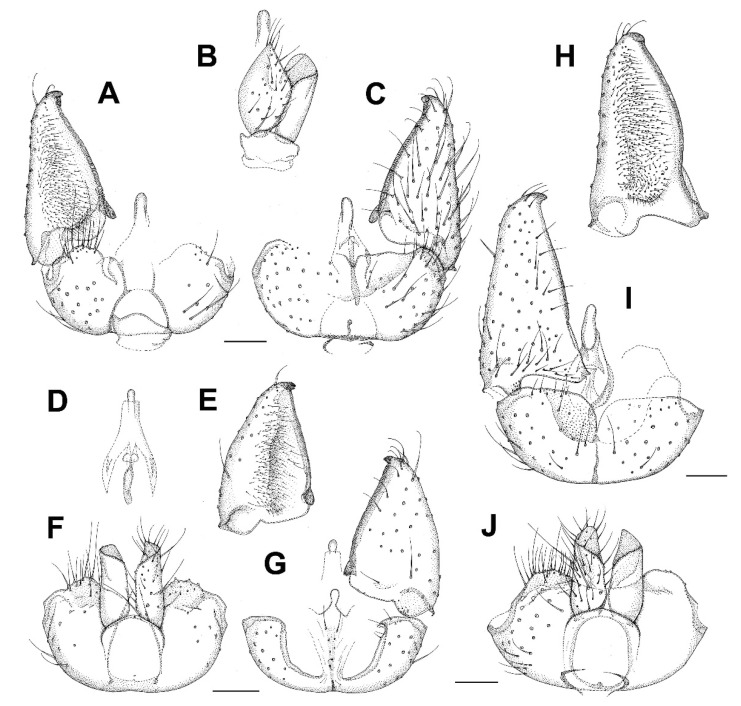
Male terminalia of *Asioditomyia* Saigusa. *Asioditomyia bruneicola* sp. nov. (holotype): (**A**) dorsal view, (**B**) cerci, (**C**) ventral view; *Asioditomyia lacii* sp. nov. (holotype), (**D**) aedeagus in ventral view, (**E**) gonostylus in dorsal view, (**F**) dorsal view with cerci, (**G**) ventral view; *Asioditomyia japonica* (Sasakawa), (**H**) dorsal view of gonostylus, (**I**) ventral view, (**J**) dorsal view with cerci. Scale bars: 0.1 mm.

***Asioditomyia bruneicola* sp. nov.** (Figure 8A–C).

LSID urn:lsid:zoobank.org:act:6F7AC59C-F435-466C-80EC-215C5C303774.

**Type material.** Holotype: male, Brunei, Ulu Temburong National Park, Kuala Belalong Field Studies Centre, 10.–12.1.2014, Malaise trap (G2M), J. Ševčík leg. (specimen after DNA extraction, No. JSD5, mounted on slide in Euparal), in coll. UBDC. Paratype: male, the same data as holotype, except for 7.–12.1.2014, Malaise trap (MT3), specimen in ethanol, after DNA extraction (No. JSD5B), in coll. JSL-OUC.

**DNA sequences.** DNA sequences (28S and COI barcode region) taken from the holotype (No. JSD5) are deposited in GenBank. Their Accession numbers are in Table S1.

**Etymology.** The specific is name is derived from Brunei Darussalam, where the type material was collected.

**Diagnosis.** This species is very similar to *Asioditomyia lacii* sp. nov. and *A. japonica* (Sasakawa, 1963). Male terminalia of *A. japonica* (specimen recently collected in Japan) are figured here for comparison (Figure 8H–J). All the three species differ slightly in the shape of gonostylus (more elongated in *A. bruneicola* sp. nov. and *A. japonica*) and tergite 9 (shorter in *A. bruneicola* sp. nov.). *A. japonica* is also distinctly larger (wing length 4.5 mm), with vein R_2+3_ longer.

**Description.** Male. Mostly brown, with yellowish markings. Wing length 3.3 mm. Head. Light brown with short black setae, areas around ocelli blackish. Three ocelli, closely set to each other, median ocellus smaller. Frontal suture present. Compound eye semiglobular, not emarginated around antennal base. Maxillary palpus yellowish brown, consisting of one segment. Antennae with 15 flagellomeres, F15 min, tapering. Flagellomeres mostly light brown with short dark setae. Scape and pedicel dark brown. Bases of antennal setae marked with a black spot. Thorax. Yellowish, with dark longitudinal stripes on sides and scutum. Mediotergite dark brown. Lateral pleura and mediotergite bare. Legs. Mostly dark brown. Coxae yellowish, apically darker. Legs covered with microtrichia and sparse setae. Tibial spurs 1:2:2. Wing hyaline, with macrotrichia on membrane. C only slightly produced, ending about one-fifth the distance between R_4+5_ and M_1_. Sc short, ending free, followed by long, faint, fold line, ending free near level of base of Rs. R_2+3_ long, its base basal to level of medial fork. R-m absent, the veins meeting in one point. Stem of M weak. CuA weak, setose, reaching wing margin. Abdomen mostly dark brown, with yellow basal bands on tergites 2–7. Terminalia (Figure 8A–C). Tergite 9 small, subrectangular, cerci broad, flap-like. Gonocoxites fused, relatively short, about twice as broad as long. Gonostylus broad, more than twice as long as broad, D-shaped, tapering posteriorly, with an elongate black sclerotized process on inner edge, which is lined by numerous closely set short dark setae. Female. Unknown.

***Asioditomyia lacii* sp. nov.** (Figure 8D–G).

LSID urn:lsid:zoobank.org:act:9BB2FE2E-E68C-480C-AF95-FC912F9C9953.

**Type material.** Holotype: male, Taiwan (China), FuShan, botanical garden, 9.v.2018, J. Ševčík leg., car netting (specimen after DNA extraction, No. JSTW38), in coll. NMNS.

**DNA sequences.** DNA sequences (28S and COI barcode region) taken from the holotype (No. JSTW38) are deposited in GenBank. Its Accession number is in Table S1.

**Etymology.** This species is named after László Papp (1946–2021) for his achievements in the study of Oriental Diptera. He was an excellent Hungarian dipterist and a very friendly man, who inspired the first author of this paper to study Taiwanese Diptera. The specific name is derived from “Laci”, a domestic form of the name László.

**Diagnosis.** This species is very similar to *Asioditomyia bruneicola* sp. nov. and *A. japonica* (Sasakawa, 1963). The gonostylus is slightly less elongated than in *A. bruneicola* sp. nov. and *A. japonica*, and tergite 9 is longer than in *A. bruneicola* sp. nov.

**Description.** Male. Mostly brown, with yellowish markings. Wing length 3.1 mm. Head. Light brown, areas around ocelli blackish. Three ocelli, in a transverse row, median ocellus smaller. Compound eye not emarginated at antennal base. Maxillary palpus yellowish brown, with one visible basal segment. Antennae with apical flagellomeres missing. Flagellomeres mostly light brown with short dark setae. Scape and pedicel dark brown. Thorax. Yellowish, with dark longitudinal stripes on sides and scutum. Mediotergite dark brown. Lateral pleura and mediotergite bare. Legs. Mostly dark brown. Coxae yellowish, apically darker. Legs covered with microtrichia and sparse setae. Tibial spurs 1:2:2. Wing the same as in *A. bruneicola* sp. nov. Abdomen mostly dark brown, with yellow basal bands on tergites 2–4. Terminalia (Figure 8D–G). Tergite 9 as long as wide, cerci flap-like. Gonocoxites fused, relatively short, about twice as broad as long. Gonostylus broad, less than twice as long as broad, D-shaped, tapering posteriorly, with a short black sclerotized process on inner edge, which is lined by numerous closely set short dark setae. Female. Unknown.

#### 3.4.2. *Burmasymmerus* gen. nov.

LSID urn:lsid:zoobank.org:act:C4D10364-3C96-44D5-B756-E0E9B4BF485C.

**Type species.***Burmasymmerus korneliae* sp. nov.

**Gender.** Masculine.

**Etymology.** The generic name is derived from ”Burma“ (=Myanmar), the country of the origin of the amber, and ”*Symmerus*“, a similar extant genus of Ditomyiidae.

**Diagnosis.** Wing length approximately 1.8 mm. Wing venation similar to that of *Symmerus* Walker, 1848. Wing relatively broad, with ratio of length to width about 2.5. Macrotrichia on wing membrane restricted to the distal third of the wing. Vein R_2+3_ relatively short, about half as long as R_5_, r-m present or absent, CuP and anal vein weak. Antennae with 14 or 15 laterally compressed flagellomeres. Male terminalia with gonostyli as long as gonocoxites, narrow, slightly expanded distally and apically rounded.

**Discussion.** The placement of this genus in Ditomyiidae is based on the following characters: vein R_2+3_ present and relatively long (about half as long as R_5_), Sc ending free and apically weak, R_4_ absent, r-m fusion absent, stem of M-fork relatively long (more than half of M_1_), macrotrichia on wing membrane present. Concerning the wing venation, the new genus is most similar to the extant genus *Symmerus* Walker, differing in the considerably smaller body size, shorter wings, shorter R_2+3_, the absence of macrotrichia in basal half of wing, vein CuP not reaching wing margin, and simpler structure of the male terminalia.

The family Ditomyiidae has not been recorded from the Burmese amber previously, neither from any other Mesozoic amber or deposits. It is, thus, the oldest geological record of the family. Ansorge [24] established a new family Eoditomyiidae from the lower Jurassic of Germany, including only the type genus *Eoditomyia* Ansorge, 1996, but this genus differs from *Burmasymmerus* gen. nov. mainly in the presence of both R_2+3_ and R_4_.

**Figure 9:**
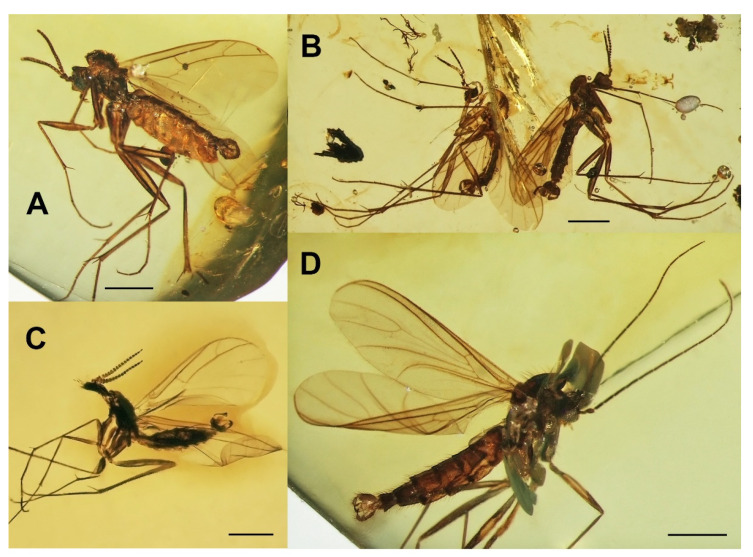
Habitus photographs of new fossil taxa. (**A**) *Burmasymmerus korneliae* gen. et sp. nov. (holotype, No. MP/4342); (**B**,**C**). *Burmasymmerus wieslawi* gen. et sp. nov. (**B**), holotype No. MP/4341/1 on the left and paratype No. MP/4341/2 on the right, in syninclusion, (**C**), paratype No. 154/2020; (**D**) *Burmatricha mesozoica* gen. et sp. nov. (holotype, No. 118/2020). Scale bars: 1 mm.

**Figure 10:**
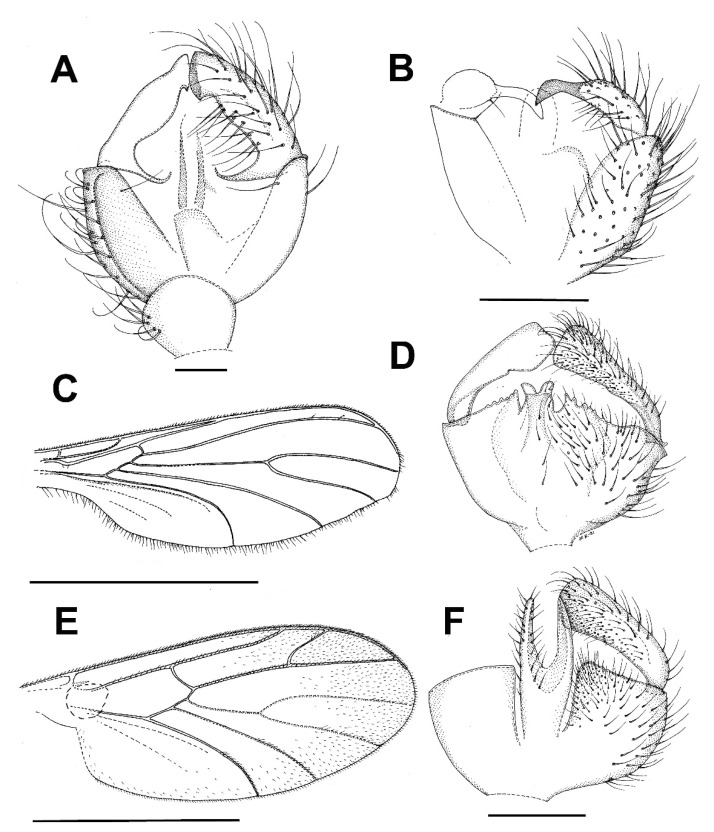
Male terminalia and wings of new fossil taxa. (**A**,**E**). *Burmasymmerus korneliae* gen. et sp. nov. (holotype, (**A**) male terminalia and (**E**) wing); (**B**,**C**). *Burmatricha mesosoica* gen. et sp. nov. (holotype, (**B**) male terminalia and (**C**) wing); (**D**,**F**). *Burmasymmerus wieslawi* gen. et sp. nov. (holotype, male terminalia, (**D**) ventral view, (**F**) dorsal view). Scale bars: 0.1 mm (terminalia), 1 mm (wings).

***Burmasymmerus korneliae* sp. nov.** (Figure 9A and Figure 10A,E).

LSID urn:lsid:zoobank.org:act:99CE3A52-BB9E-46A1-9730-2653ED281949.

**Type material.** Holotype: male, No. MP/4342, Burmese amber (the earliest Cenomanian, 98.79 ± 0.62 Ma), in coll. ISEA PAS.

**Etymology.** This species is named after Dr. Kornelia Skibińska (Institute of Systematic and Evolution of Animals, Polish Academy of Sciences, Kraków) for her achievements in the study of fossil Diptera.

**Diagnosis.** This species is easily recognizable by its relatively long crossvein r-m, typical of the extant genus *Symmerus*, and by the 14-segmented antennal flagellum. Further diagnostic characters are the long, mediobasal projection of the gonostylus, subapically constricted gonostylus, and the shape of the medioventral process of gonocoxites.

**Description.** Wing length 1.8 mm. Body all dark brown. Head. Three ocelli placed on a frontal tubercle, median ocellus much smaller. Compound eye oval, emarginated at antennal base, forming an incomplete bridge above antennae. Palpi with three segments. Antennae with 14 flagellomeres, about as long as wide, laterally compressed. Scutum and scutellum with long dense setae. Thoracic pleura and mediotergite bare. Coxae dark brown, rest of legs light brown. Legs with dense microtrichia. Several sparse posterior setae on mid and hind tibia, shorter than maximum tibial diameter. Tibial spurs 1:2:2. Wing hyaline, its membrane covered with microtrichia, plus sparse macrotrichia in distal half of the wing. C distinctly produced, ending about one-third the distance between R_4+5_ and M_1_. Sc short, ending free, followed by long faint fold line. R_2+3_ about half as long as R_5_. R-m present, as long as m-m. Stem of M-fork weak. CuP long but weak, setose, reaching wing margin. Abdomen dark brown. Terminalia (Figure 10A,E) with tergite 9 short, about as long as broad, cerci longer than tergite 9, narrow and apically pointed. Gonocoxites fused, with the medioventral process narrow, forked, longer than gonocoxite. Gonostylus as long as gonocoxite, with distinct ventrobasal projection and subapical constriction. Posterior margin of gonostylus rounded.

***Burmasymmerus wieslawi* sp. nov.** (Figure 9B,C and Figure 10D,F).

LSID urn:lsid:zoobank.org:act:F39AA90B-801D-4702-8EDA-C0B58E2C6015.

**Type material.** Holotype: male, No. MP/4341/1, Burmese amber (the earliest Cenomanian, 98.79 ± 0.62 Ma), in coll. ISEA PAS. Paratypes (all Burmese amber inclusions): male No. MP/4341/2 (in the same piece of amber as holotype, ISEA PAS), male No. 154/2020 (JSL-UOC), male No. 176/2020 (SMOC), male No. 404/2019 (JSL-UOC).

**Etymology.** This species is named after Prof. Wiesław Krzemiński (Institute of Systematic and Evolution of Animals, Polish Academy of Sciences, Kraków) for his achievements in the study of fossil Diptera.

**Diagnosis.** This species differs from *B. korneliae* sp. nov. mainly by 15-segmented antennal flagellum, the absence of crossvein r-m, and in the shape of medioventral process of gonocoxites.

**Description.** Wing length 1.6 mm (holotype). Body all dark brown. Head. Three ocelli placed on a frontal tubercle, median ocellus much smaller. Compound eye oval, not distinctly emarginated at antennal base, bridge above the antennae not discernable. Palpi with three visible segments. Antennae with 15 flagellomeres, about as long as wide, laterally compressed, the apical flagellomere narrow, about half as broad as flagellomere 14. Scutum and scutellum with long dense setae. Thoracic pleura and mediotergite bare. Coxae and femora dark brown, the rest of legs brown. Legs with dense microtrichia. Several sparse posterior setae on mid and hind tibia, shorter than maximum tibial diameter. Tibial spurs 1:2:2. Wing hyaline, its membrane covered with microtrichia, plus sparse macrotrichia in distal half of the wing. C distinctly produced, ending slightly more than one-third the distance between R_4+5_ and M_1_. Sc short, ending free, followed by long faint fold line. R_2+3_ about half as long as R_5_. R-m absent. Stem of M-fork weak. CuP long but weak, setose, reaching wing margin. Abdomen dark brown. Terminalia (Figure 10D,F) with tergite 9 short, about as long as broad, cerci much longer than tergite 9, strongly sclerotized, with branches apically pointed. Gonocoxites fused, with the medioventral process narrow, relatively short, apically with a shallow depression, not distinctly forked. Gonostylus as long as gonocoxite, without any ventrobasal projection and without distinct subapical constriction.

**Discussion.** As mentioned in the Diagnosis above, this species is characteristic with its 15-segmented antennal flagellum and the absence of crossvein r-m. Both these character states are typical also of the extant genus *Asioditomyia*, see above, which differs from *Burmahesperinus* gen. nov. mainly by the much longer vein R_2+3_, completely setose wing, and broader gonostyli. However, we consider as premature to establish here a separate genus for *B. wieslawi* sp. nov., until more specimens are available and the real diversity of Burmese amber Ditomyiidae is better known.

#### 3.4.3. Genus *Celebesomyia* Saigusa, 1973

A monotypic genus, with only one species hitherto described [23]. Here, we present a figure of the male terminalia of this species for the first time. This genus differs from the other genera of Ditomyiidae mainly in the absence of ocelli.

**Figure 11:**
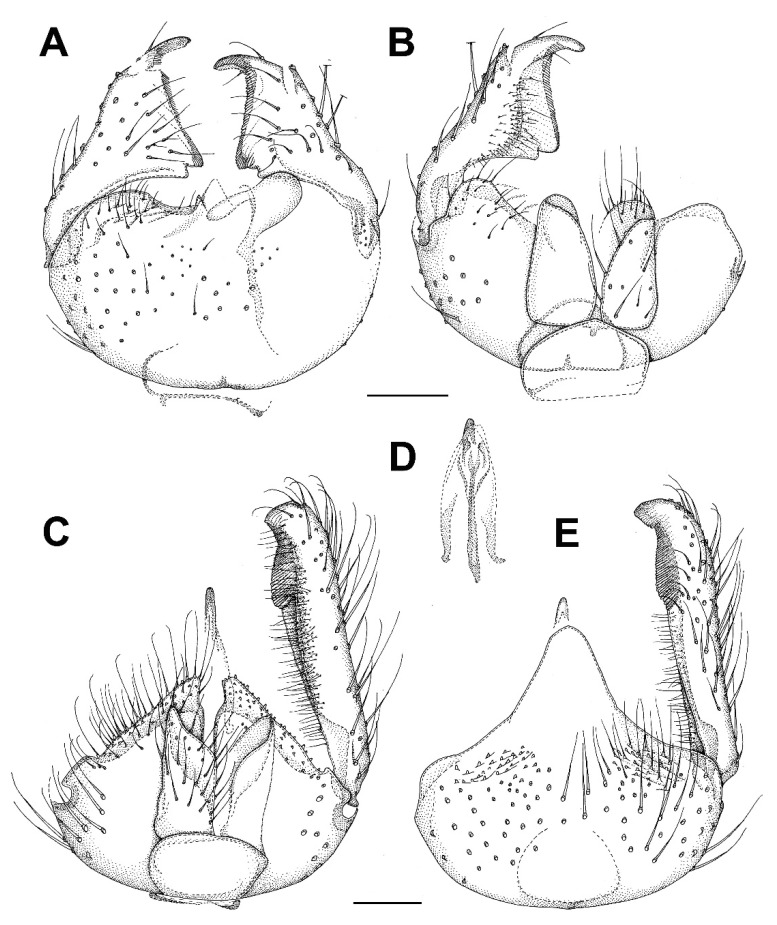
Male terminalia of Ditomyiidae. *Celebesomyia inocellata* Saigusa, (**A**) ventral view, (**B**) dorsal view; *Ditomyia asiatica* sp. nov. (holotype), (**C**) dorsal view with cerci, (**D**) aedeagus in ventral view, (**E**) ventral view. Scale bars: 0.1 mm.

***Celebesomyia inocellata* Saigusa,** 1973 (Figure 11A,B).

**Material examined.** Indonesia, South Sulawesi, Bulusaraung, 1066 m, 18.–27.viii.2007, Malaise trap No. INDO708M1B (MNHN, specimen after DNA extraction, No. JSD8, partly damaged, mounted on slide in Euparal).

**DNA sequences.** DNA sequences (28S and COI barcode region) taken from the specimen No. JSD8 are deposited in GenBank. Their Accession numbers are in Table S1.

**Figure 12:**
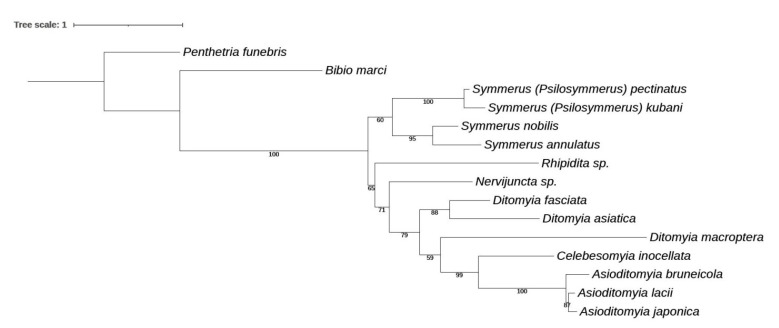
Phylogenetic relationships (Maximum Likelihood hypothesis) among the species of Ditomyiidae based on two DNA markers (28S and COI).

**Discussion**. This species was described based on a single female collected in Central Sulawesi [23], and no corresponding male has been known up to the present. The figure of the male terminalia (Figure 11A,B) shows its rather similar structure to *Asioditomyia* species. Species of *Asioditomyia* and *Celebesomyia* form a monophyletic group also in the molecular tree of Ditomyiidae, based on 28S and COI gene fragments (Figure 12), confirming that these two genera are closely related, if not identical. As noted already by Saigusa [23], *Celebesomyia* “most resembles the genus *Asioditomyia* in every structure” and “is almost certainly an offspring of the extinct *Asioditomyia* species which invaded into Celebes from the South Eastern Asia”. The generic concept of Ditomyiidae has not been fully resolved yet, as recently commented on by Fitzgerald [25], and further phylogenetic studies, covering all the genera in this family, may change current classification.

#### 3.4.4. Genus *Ditomyia* Winnertz, 1846

Type genus of the family Ditomyiidae, with 11 species currently described from the Holarctic region, and one from Guatemala [25]. An additional species is described below.

***Ditomyia asiatica* sp. nov.** (Figure 3C and Figure 11C–E).

LSID urn:lsid:zoobank.org:act:7A75CF9D-DD86-45F9-B1F8-B8CA582859C3.

**Type material.** Holotype: male. Thailand, Chiang Mai, Doi Inthanon National Park, 177-2014 (without other data), coll. QSBG (permanent preparation in Euparal, specimen after DNA extraction, No. JSBA46). Paratypes (4 males). 2 males with the same data as holotype (JSL-UOC: specimen after DNA extraction, No. JSBA46D, SMOC). 2 males: Thailand, Chiang Mai, Doi Inthanon NP, Kew Mae Pan, 18°33.163′ N, 98°28.8′ E, 2200 m, 22.vii-2.viii.2006, Y. Areeluck leg., Malaise trap (T120), coll. QSBC.

**DNA sequences.** DNA sequences (28S and COI barcode region) taken from the holotype No. JSBA46 and one paratype (No. JSBA46D) are deposited in GenBank. Their Accession numbers are in Table S1.

**Etymology.** The specific is name is derived from Asia, referring to the continent where this species occurs.

**Diagnosis.** This species is characteristic with its dark coloration, narrow gonostyli, and large medioventral lobe of gonocoxites.

**Description.** Male (Figure 3C). Mostly dark brown. Wing length 4.5 mm. Head. Dark brown with short black setae, areas around ocelli blackish. Three dorsomedial ocelli, arranged in a transverse line, median ocellus much smaller. Frontal suture present. Compound eye semiglobular, not emarginated at antennal base. Maxillary palps brown, two-segmented with basal segment stout, and apical segment smaller. Antennae with 15 flagellomeres, F15 min, tapering. Flagellomeres mostly light brown with short dark setae. Pedicel and scape brown. Bases of antennal setae marked with a black spot. Thorax. All uniformly dark brown with mediotergite lighter. Scutum with long dark setae laterally and in dorsocentral rows. Scutellum with a row of subapical setae. Thoracic pleura and mediotergite bare. Legs. Coxae dark brown, the rest of legs light brown. Legs with dense microtrichia. A row of sparse black posterior setae running nearly full length of hind tibia. Tibial spurs 1:2:2. Wing hyaline and darkened, with macrotrichia on the membrane, but mostly concentrated near the wing tip and front margin. C ending about one-fourth the distance between R_4+5_ and M_1_. Sc short, ending free, followed by long, faint, fold line, ending free near level of base of Rs. R_2+3_ long, base basal to level of medial fork. R-m present but short. Stem of M weak. Anal vein long, setose, reaching wing margin. Abdomen. Mostly dark brown, with yellow thin posterior bands on tergites 2–4. Terminalia (Figure 11C–E). Tergite nine short, subrectangular. Cerci twice as long as T9, flap-like. Gonocoxites fused ventrally, with posterior margin developed into a distinct median lobe. Gonostylus elongate, narrow, 5 times as long as broad, apically rounded, posterior part of its inner edge lined by numerous closely set short dark setae. Female. Similar to male in most aspects.

### 3.5. Family Keroplatidae

This is a relatively large and diverse family of Sciaroidea, currently comprising more than 1000 species in almost 100 genera and 6 subfamilies, including the subfamily Lygistorrhininae, previously considered as a separate family [3,11]. Oriental species of Keroplatidae were recently studied mostly by L. Papp and J. Ševčík (e.g., [26,27,28]). Several new extant species are described below.

#### 3.5.1. Genus *Chetoneura* Colless, 1962

A small and peculiar genus of uncertain phylogenetic position [3], with 3 species currently described, all from the Oriental region [29]. An additional new species is described below.

**Figure 13:**
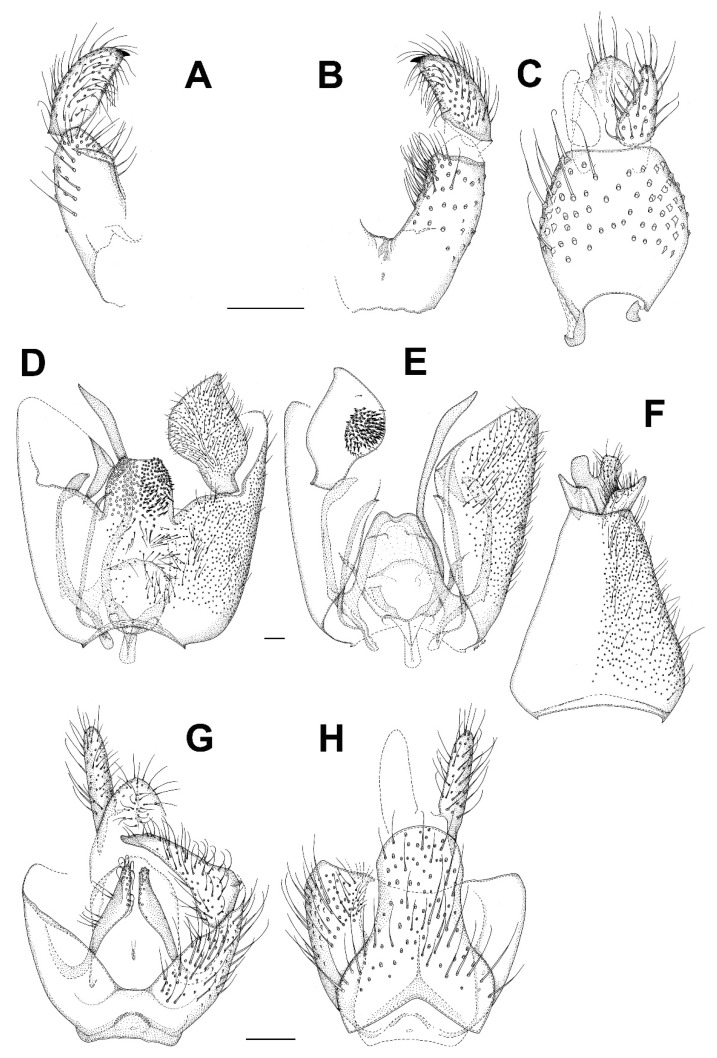
Male terminalia of new species of Keroplatidae. *Chetoneura davidi* sp. nov. (holotype), (**A**) dorsal view,(**B**) ventral view, (**C**) tergite 9; *Euceroplatus mantici* sp. nov. (holotype), (**D**) ventral view, (**E**) dorsal view, (**F**) tergite 9; *Platyceridion yunfui* sp. nov. (holotype), (**G**) ventral view, (**H**) dorsal view. Scale bars: 0.1 mm.

***Chetoneura davidi* sp. nov.** (Figure 13A–C).

LSID urn:lsid:zoobank.org:act:F573CB97-9772-4A3B-B284-F227533BD597

**Type material.** Holotype: male, Brunei, Ulu Temburong National Park, Kuala Belalong Field Studies Centre, 26.i.–15.ii.2015, Malaise trap No. IDD, D. Kaspřák & M. Mantič leg. (specimen after DNA extraction, mounted in Euparal, No. JSK59, coll. UBDC). Paratype: male, the same data as holotype, except 12–14.i.2014, Malaise trap No. R1M, D. Kaspřák & J. Ševčík leg. (coll. JSL-UOC).

**DNA sequences.** DNA sequences (COI barcode region) taken from the holotype (No. JSK59) and paratype (No. JSK59B) are deposited in GenBank. Their Accession numbers are in Table S1.

**Etymology.** This new species is named after Dr. David Kaspřák, a former doctoral student of the first author of this paper. He participated at the field research in Brunei and collected the type specimens of this species.

**Diagnosis.** This species is most similar to the type species of the genus, *Chetoneura cavernae* Colless, 1962, sharing principally the same wing venation with very short r-m fusion, and also similar structure of the male terminalia, with an apical tooth on the apex of gonostylus. The other two species of *Chetoneura* Colless, 1962 (=*Bisubcosta* Papp, 2006) have substantially longer r-m fusion [29]. *Chetoneura davidi* sp. nov. differs from *Ch. cavernae* mainly in the overall body coloration (thorax, coxae and abdomen partly yellowish and knob of haltere dark), costa less produced beyond the tip of R_5_, and relatively narrow gonostylus.

**Description.** Male. Wing length 4.6 mm (holotype). Head. Compound eyes relatively broad, covering most of the head in lateral view. Two ocelli (paratype, ocelli absent in holotype). Face broad (about as high as broad), brown, setose in its lower half, weakly sclerotized. Clypeus setose. Mouthparts reduced. Maxillary palpus consists of a small yellowish palpifer and an oval dark palpomere. Antenna long, about 1.5 times as long as the head and thorax together, with 14 flagellomeres. Flagellum laterally flattened, flagellomeres slightly prolonged both anteriorly and posteriorly. All flagellomeres dark brown. Scape and pedicel dark brown, slightly shorter than wide. Scutum yellowish, with lateral margins and two submedian longitudinal stripes dark (V-shaped, connecting posteriorly). Scutellum dark brown, medially darker, with subapical transverse row of short black setae. Mediotergite brown, medially setose, posteriorly only slightly protruding. Subscutellar membranous area very thin and indistinct, transverse, not tapering posteriorly. Lateral sclerites yellowish, only anepisternum dark. Laterotergite bare. Antepronotum and proepisternum dark and setose. Anterior spiracle and membranous area around it without setae. Anepisternum setose in its upper half, the other lateral sclerites bare. Haltere with the knob dark brown and stem lighter. Wing hyaline, without markings. Sc relatively long, reaching beyond r-m fusion. Vein C produced beyond R_5_ to about one third of the distance between the tips of R_5_ and M_1_. R_2+3_ absent. M_2_ reaching wing margin. CuA downcurved in the middle but almost straight towards the tip. A_1_ short, not reaching wing margin. Costa, R_1_ and R_5_ covered with setae. Legs mostly yellowish brown, with coxae lighter but apically darker. Femora all dark. Tibiae brown. All tibiae with trichia arranged in dense longitudinal rows, without strong bristles. Fore tibia without distinct apical spur. Only one spur present on both mid and hind tibia. Spur on hind tibia rather long, about four times as long as maximum (apical) tibial diameter. A distinct transverse comb of closely set posterior setulae present apically on each tibia. Abdomen relatively long, bicolored, with segments 1–5 mostly yellowish, with dark posterior bands. Segments 6–9 all dark. Terminalia (Figure 13A–C) dark brown. Tergite 9 slightly longer than broad. Gonocoxites broadly fused ventrobasally. Gonostylus shorter than gonocoxite, relatively narrow, about twice as long as wide, with a distinct apical tooth.

**Discussion.** The absence of ocelli in the holotype is noteworthy (in the paratype male two ocelli are present), and undoubtedly represents an individual aberration, rather than a useful taxonomic character. Both the holotype and paratype have identical COI barcode sequences.

#### 3.5.2. Genus *Euceroplatus* Edwards, 1929

A small genus, with 9 described species from the Oriental or Australasian regions [29]. All these species have a species-specific color pattern on wings. An additional new species is described here. This species was included in the molecular analysis by Mantič et al. [3], under the name *Euceroplatus* sp. 1, and proved to be closely related to the more typical species of the genus, though undescribed, referred to as *Euceroplatus* sp. 2.

***Euceroplatus mantici* sp. nov.** (Figure 3D and Figure 13D–F).

LSID urn:lsid:zoobank.org:act:3E5DD9BE-BA08-48B8-BEEF-1A252DF538DF.

**Type material.** Holotype: male, Thailand, Chiang Mai, Ban Mae Lao, 24.v.2017, sweeping vegetation, M. Mantič leg. (SMOC).

**DNA sequences.** DNA sequence (COI barcode region) taken from the holotype (specimen No. JSBA24) is deposited in GenBank. Its Accession number is provided in Table S1.

**Etymology.** This distinct new species is named after Dr. Michal Mantič, a former doctoral student of the first author of this paper, who collected the holotype. He participated also at the field trip to Brunei, where some other species included in this paper were collected.

**Diagnosis.** This species is rather unique and unmistakable due to its robust habitus, resembling rather some species of *Keroplatus* Bosc, 1792, than those of *Euceroplatus*. Further diagnostic characters are on the wing (central spot and subapical dark band), and a relatively broad gonostylus, placed rather dorsally than posteriorly on the gonocoxite, without an apical tooth or long setae.

**Description.** Male. Body robust, mostly yellowish brown. Wing length 5.0 mm. Head. Mostly brown, with dark short trichia beyond the eyes and on vertex. Three ocelli, the median one smaller, the distance between the eye margin and lateral ocellus is about the diameter of the latter. Ocelli placed on a dark triangle, connecting the eyes and the posterior margin of the head. Face narrow (three times as high as broad), yellowish, bare, weakly sclerotized and in its upper half medially divided by a dark sagittal furrow. Clypeus small and indistinct. Mouthparts reduced. Maxillary palpus consists of a small palpifer and a larger oval yellowish palpomere. Antenna relatively short, about 2.2 times as long as the head, with 14 flagellomeres. Flagellum laterally strongly flattened. All flagellomeres dark brown. Both F11 and F14 partly lighter (less than half of the flagellomere). Scape and pedicel dark brown, each of them twice as wide as long. Thorax. Scutum weakly arched, evenly covered with short setae and with longer setae along lateral margins, mostly yellowish brown, with dark posterolateral corners and two thin dark submedian longitudinal stripes (V-shaped, connecting posteriorly). Scutellum yellowish brown, dorsally densely covered with short black setae, without long apical bristles. Mediotergite yellowish, laterally brown, bare, posteriorly only slightly protruding. Subscutellar membranous area almost indistinct, reduced to a narrow transverse band. Laterotergite bare, yellowish, with darker upper and lower parts. Antepronotum and proepisternum densely setose. Anterior spiracle and membranous area around it without setae. Anepisternum bare, yellowish, with anterior third dark. Prosternum densely setose. Haltere bicoloured, with the stem lighter and the knob laterally dark. Wing (Figure 3D) hyaline, with a large central spot and a broad subapical band. Sc rather long, setose, reaching beyond the base of r-m fusion. Vein C produced beyond R_5_ to slightly more than a quarter of the distance between the tips of R_5_ and M_1_. R_2+3_ ending in C, almost touching the apex of R_1_. All median branches shortened, not reaching wing margin. CuA almost straight, not distinctly downcurved. A_1_ strong, ending just before the wing margin. Costa, Sc, r-m fusion, R_1_ and R_5_ covered with strong setae. Legs mostly yellowish brown, tibiae and tarsi slightly lighter. Coxae mostly yellowish, darkened at apex, densely covered with dark setae along their anterolateral (C1, C2) or posterolateral (C3) surface. Femora mostly yellowish brown, evenly covered with short dark setulae. Tibiae light brown, darkened at apical ends. All tibiae with trichia arranged in dense longitudinal rows, with several scattered stronger setae. The apex of fore tibia without any distinct tibial organ. Fore tibia with one apical spur, slightly longer than maximum tibial diameter. Two spurs present on both mid and hind tibia, the posteroventral spurs about twice as long as the anteroventral ones. A distinct transverse comb of closely set posterior setulae apically on mid and hind tibia. Claws relatively large, simple, with short dense setulae basally. Abdomen bicolored, mostly ochreous, with tergites 5–7 darkened. Terminalia (Figure 13D–F) ochreous, posteriorly darker. Tergite 9 subtriangular, slightly longer than broad. Gonocoxites fused ventrally, with a distinct medioventral lobe, covered with short black setae. Gonostylus less than half the length of gonocoxite, subrectangular to oval in shape, dorsally with a patch of short black setae.

#### 3.5.3. Genus *Platyceridion* Tollet, 1955

Only two species of *Platyceridion* have been described up to the present, both from Sri Lanka, and they are known to have mycmecophilous larvae [30]. An additional species is described here form the Hainan Island, confirming a broader distributional pattern of the genus and possible association with islands. The biology of the new species remains unknown, although myrmecophily cannot be excluded due to common occurrence of an unidentified species of ants at the type locality.

***Platyceridion yunfui* sp. nov.** (Figure 3E and Figure 13G,H).

LSID urn:lsid:zoobank.org:act:9C315405-B814-4E29-8A97-3D397DDF6918.

**Type material.** Holotype: male, China, Hainan, Semangat Gunung, 1300 m, 22.v.2016, Yunfu Chen & Jan Ševčík leg. (SMOC). Paratype: female, the same data as the holotype, except for 21.v.2016 (JSL-OUC).

**DNA sequence.** DNA sequence (COI barcode region) taken from the holotype (No. JSK81A) is deposited in GenBank. Its Accession number is provided in Table S1.

**Etymology.** This species is named after Dr. Yunfu Chen, a former student at the Sun Yat-sen University in Guangzhou, China. He participated at the field trip to Hainan Island, where this species was collected.

**Diagnosis.** *Platyceridion yunfui* sp. nov. is most similar to *P. talaroceroides* (Senior-White, 1921), known from Sri Lanka [30], mainly due to the same coloration of the abdomen but differs in the structure of flagellum and in minor details on the male terminalia. The new species possesses long projections on flagellomeres 1 to 13 (1 to 12 in *P. talaroceroides*), its tergite 9 is somewhat broader and cerci longer than half of the length of T9. In addition, the gonostylus appears longer and less pointed apically than in *P. talaroceroides*. *P. edax* Chandler and Matile differs in the entirely yellow tergites 1–5.

**Description.** Male. Wing length 3.2 mm (holotype). Head yellowish brown, with dark setae posteriorly. Three dark ocelli, closely set to each other, the median one much smaller, the distance between the eye margin and lateral ocellus is about 1.5 times the diameter of the latter. Face twice as broad as high, yellowish, bare. Clypeus circular, covered with dark setae. Maxillary palpus brownish, consists of a small palpifer and three short palpomeres. Basal palpomere with a distinct sensory pit on its upper surface. Antenna about as long as thorax, strongly pectinate, with 14 flagellomeres. Flagellomeres with long anterior projections, almost as long as flagellum. F1 basally yellowish, the rest of flagellum brown. Both scape and pedicel yellowish, shorter than wide, with a ring of short dark setae. Thorax. Mostly brownish yellow, with scutellum and mediotergite dark. Scutum weakly arched, densely covered with dark setae. Scutellum dark brown, with short setae dorsally and a row of longer black setae subapically. Mediotergite brown, basally lighter, bare, posteriorly sharply protruding. Subscutellar membranous area indistinct. Lateral sclerites mostly yellowish. Laterotergite brownish, covered with long dark setae. Antepronotum and proepisternum setose. Anterior spiracle and membranous area around it with several short setae. Anepisternum with a patch of setae along its upper margin, the other lateral sclerites bare. Haltere slightly longer than the first abdominal tergite, with knob dark brown and stem lighter. Wing (Figure 3E) hyaline, broadly darkened along distal and posterior margins and in central parts of the wing. Sc short, ending in C. Vein C produced beyond R_5_ to about one third of the distance between the tips of R_5_ and M1. R_2+3_ short, ending in C. M_2_ distinctly shortened, not reaching wing margin. CuA2 straight. A1 strong, reaching to wing margin. Costa, R_1_ and R_5_ covered with setae. Legs mostly yellowish, densely covered with dark trichia. Coxae and femora all yellowish. Tibiae light brown. All tibiae with trichia arranged in dense longitudinal rows. Fore tibia with one apical spur, two spurs present on both mid and hind tibia. The posteroventral spur on hind tibia about five times as long as the anteroventral one. A distinct transverse comb of closely set posterior setulae present on mid and hind tibia apically. Abdomen bicolored, basally yellowish brown, apically dark brown. Tergites 2–5 yellowish, with proximal half dark. Terminalia (Figure 13G,H) dark brown. Tergite 9 pear-shaped, basally broader, slightly longer than broad. Cerci long, about two thirds of the length of T9. Gonocoxites fused ventrobasally. Gonostylus about as long as gonocoxite, tapering.

Female. Similar to male, except for the antennae and wing markings. Flagellar projections shorter, at most half as long as the length of flagellum (Figure 3E). Wing markings more demarcated, with a distinct spot behind R_2+3_, behind r-m fusion and along Cu1, while the membrane around branches of M almost clear.

**Discussion.** As noted above, *Platyceridion yunfui* sp. nov. is very similar to *P. talaroceroides*, and the identity of both the species should be confirmed in the future using molecular methods.

#### 3.5.4. Genus *Setostylus* Matile, 1990

A widespread genus, with 9 species currently described from the Oriental and Neotropical regions [31]. An additional new species is described below, representing the first record of the genus from Taiwan.

**Figure 14:**
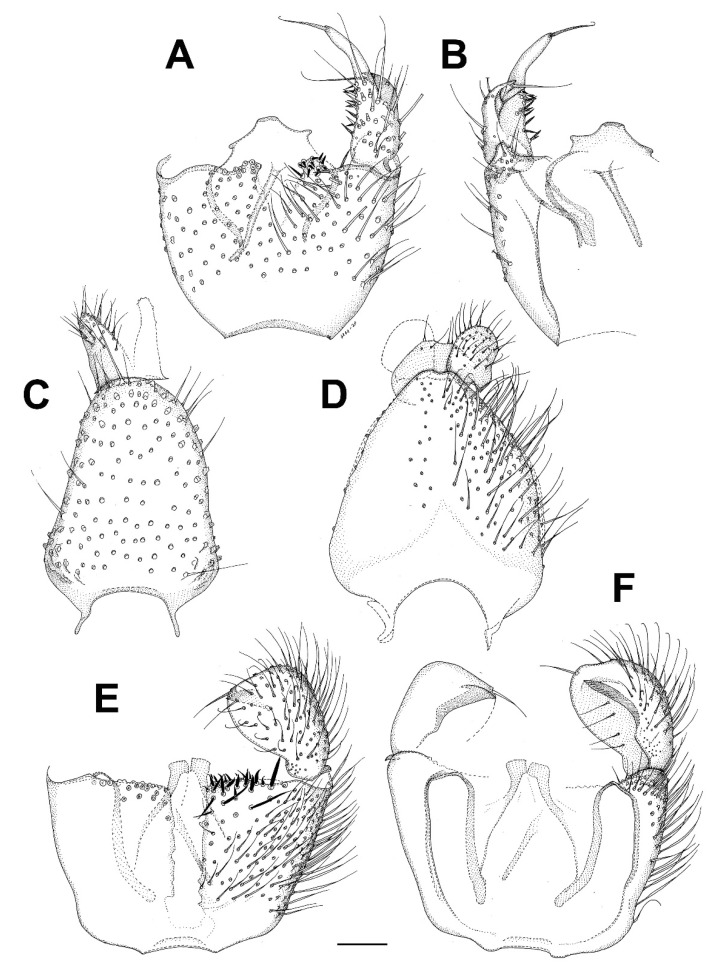
Male terminalia of new species of Keroplatidae. *Setostylus fangshuoi* sp. nov. (holotype): (**A**) ventral view, (**B**),dorsal view, (**C**) tergite 9 with cerci; *Terocelion adami* sp. nov. (holotype); (**D**) tergite 9 with cerci, (**E**) ventral view, (**F**). dorsal view. Scale bars: 0.1 mm.

**Figure 15:**
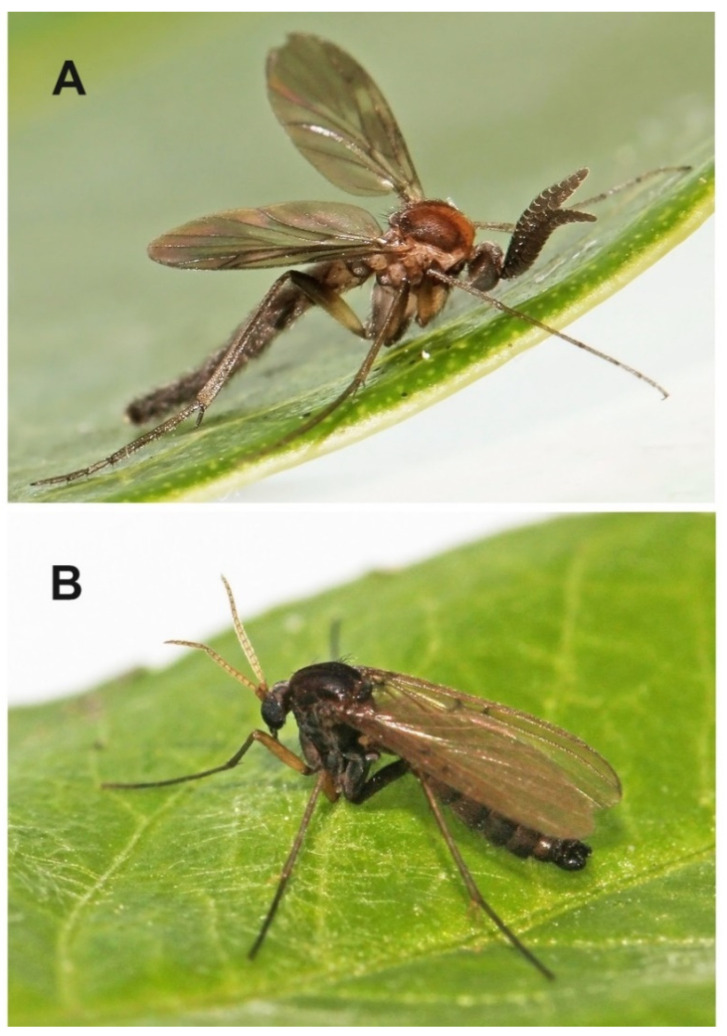
Photographs of living specimens. (**A**) *Setostylus fangshuoi* sp. nov. (holotype, male); (**B**) *Hadroneura martini* sp. nov. (holotype, male). Photo by J. Ševčík. Scale bar: 1 mm.

***Setostylus fangshuoi* sp. nov.** (Figure 14A–C and Figure 15A).

LSID urn:lsid:zoobank.org:act:24F4661A-D1AB-4FAA-BEBB-99A22610A4C7.

**Type material.** Holotype: male, Taiwan (China), FuShan Botanical Gardens, 8.–10.v.2019, at light, J. Ševčík leg. (NMNS). Paratype: male, the same data as holotype (NMPC).

**DNA sequences.** DNA sequences (COI barcode region) taken from the holotype (specimen No. JSTW36) and paratype (No. JSTW36B) are deposited in GenBank. Their Accession numbers are provided in Table S1.

**Etymology.** This species is named after Fang-Shuo Hu, a Taiwanese coleopterist and student at the National Chung Hsing University in Taichung. He participated at the field trip to FuShan Botanical gardens, where the type specimens of this new species were collected.

**Diagnosis.** This species resembles *Setostylus chinensis* Cao, Evenhuis and Zhou, 2007, described from mainland China [31], mainly in the extensively marked wings, but differs in the shape of gonostylus (basally broader in *S. fangshuoi* sp. nov.) and tergite 9 (less triangular in *S. fangshuoi* sp. nov.).

**Description.** Male. Body slender, brightly colored (Figure 15A). Wing length 2.8 mm. Head mostly brown. Compound eyes covering approximately half of the head in lateral view. Three ocelli, the median one smaller, the distance between the eye margin and lateral ocellus is about the diameter of the latter. Ocelli placed on a dark, heart-shaped tubercle. Face about as high as broad, brown, bare, weakly sclerotized and in its upper half medially divided by a dark sagittal furrow. Clypeus small and haired. Mouthparts reduced. Maxillary palpus consists of a palpifer and one palpomere, both of about the same length. Antenna relatively long, about 1.2 times as long as the head and thorax together, with 14 flagellomeres, all dark brown. Flagellum laterally strongly flattened. Flagellomeres 1–13 S-shaped, F14 subtriangular. Scape and pedicel dark brown, each of them twice as wide as long. Thorax. Scutum weakly arched, evenly covered with short setae, mostly yellowish, with two dark longitudinal stripes. Scutellum dark brown, with a subapical transverse row of short black setae, without long apical bristles. Mediotergite mostly brown, bare, posteriorly only slightly protruding. Subscutellar membranous area small and indistinct. Laterotergite bare, yellowish, with darker lower margin. Antepronotum and proepisternum setose. Anterior spiracle and membranous area around it without setae. Anepisternum setose in its uppermost part, the other lateral sclerites bare. Prosternum with sparse setae in its upper part. Haltere dark brown, basally lighter, slightly longer than the first abdominal tergite. Wing relatively broad, hyaline, extensively marked. Sc rather long, reaching to the distal end of r-m fusion. Vein C produced beyond R5 to about 3/5 of the distance between the tips of R_5_ and M_1_. R_2+3_ ending in C, almost touching the apex of R_1_. M_2_ shortened, not reaching wing margin. CuP downcurved towards the tip. A1 strong, ending just before the wing margin. Costa, R_1_ and R_5_ covered with setae. Legs. Coxa 2 and 3 mostly dark, coxa 1 lighter. Femora mostly yellowish, F2 basally darkened, F3 dark at the apical third and basally. All tibiae with trichia arranged in dense longitudinal rows, without stronger setae. The apex of fore tibia without a tibial organ. Fore tibia with one apical spur, almost twice as long as maximum tibial diameter. Two spurs present on both mid and hind tibia, the posteroventral spur on T2 about twice as long as the anteroventral one, T3 with spurs subequal in length. A distinct transverse comb of closely set posterior setulae apically on mid and hind tibia. Abdomen relatively long, mostly dark brown, tergites 2–4 basally yellowish. Terminalia (Figure 14A–C) with tergite 9 longitudinal, posteriorly rounded, not distinctly triangular. Gonocoxites fused ventrally, with short strong setae on the posterior margin, concentrated on two submedian tubercles. Gonostylus about as long as gonocoxite, broad in basal half, distally narrow, bearing a long apical seta.

#### 3.5.5. Genus *Terocelion* Ševčík, 2012

A recently described genus, similar to *Euceroplatus*, with 2 species currently described from Brunei and Thailand [28]. An additional new species is described below, representing the first record of the genus from Taiwan.

***Terocelion adami* sp. nov.** (Figure 3F and Figure 14D–F).

LSID urn:lsid:zoobank.org:act:0F3075F8-3199-45B4-AD98-7AEE072206FB.

**Type material.** Holotype: male, Taiwan (China), Nantou, Renai, 25.–28.iv.2019, Malaise trap 4, J. Ševčík & M. Tkoč leg. (NMNS). Paratype: male, the same data as holotype (SMOC).

**DNA sequences.** DNA sequence (COI barcode region) taken from the holotype (No. JSTW31) is deposited in GenBank. Its Accession number is provided in Table S1.

**Etymology.** This beautiful species is named after Adam Ševčík, the son of the first author.

**Diagnosis.** This species is most similar to *Terocelion melanoleucum* Ševčík, 2012, differing mainly in details of body coloration and the shape of gonostylus, which is distinctly shorter in the new species than in *T. melanoleucum*. The type species of the genus, *Terocelion terezae* Ševčík, 2012, is distinct with its strongly pectinate antennae [28].

**Description.** Male. Wing length 5.6 mm (holotype). Head. Compound eyes about 2.3 times as high as broad in lateral view, distinctly emarginated above the bases of antennae. Three ocelli, the median one smaller, the distance between the eye margin and lateral ocellus is less than the diameter of the latter. Ocelli placed on a dark tubercle, medially divided by a distinct frontal furrow. Face narrow (three times as high as broad), light brown, bare, weakly sclerotized and in its upper half medially divided by a dark sagittal furrow. Clypeus small and indistinct. Mouthparts reduced. Maxillary palpus consists of a small palpifer and a larger oval yellowish palpomere. Antenna as long as the head and thorax together, with 14 flagellomeres. Flagellum laterally flattened, flagellomeres 2 to 13 prolonged both anteriorly and posteriorly. F1 to F11 plus F14 dark brown, F12 and F13 white. Both F11 and F14 partly lighter (less than half of the flagellomere). Scape and pedicel dark brown, slightly shorter than wide, with dark short setae. Scutum weakly arched, evenly covered with short setae, laterally and posteriorly with longer setae, yellowish brown with lateral margins and two submedian longitudinal stripes dark (V-shaped, connecting posteriorly). Scutellum light brown, medially darker, with subapical transverse row of short black setae. Mediotergite brown, proximally lighter, bare, posteriorly distinctly protruding. Subscutellar membranous area relatively small, subtriangular, transverse, not tapering posteriorly. Lateral sclerites bicoloured, light and dark brown (Figure 3F). Laterotergite bare, with the upper half dark and the lower one light. Antepronotum and proepisternum setose. Anterior spiracle and membranous area around it without setae. Anepisternum and the other lateral sclerites bare. Prosternum without setae. Haltere slightly longer than the first abdominal tergite, with the knob dark brown and stem lighter. Wing (Figure 3F) hyaline, distinctly marked. Sc short, reaching to the base of r-m fusion. Vein C produced beyond R_5_ to slightly less than half the distance between the tips of R_5_ and M_1_. R_2+3_ ending in C. M_2_ shortened, not reaching wing margin. CuP downcurved towards the tip. A_1_ strong, ending just before the wing margin. Costa, R_1_ and R_5_ covered with setae. Legs mostly yellowish white, tibiae and tarsi slightly lighter. Coxa 1 white, coxa 2 dark on apical ¼, costa 3 dark on apical 1/3. Femora yellowish white, femur 2 dark basally, femur dark on basal 1/3. Tibiae light brown, darkened at apical ends. All tibiae with trichia arranged in dense longitudinal rows, without strong bristles. The apex of fore tibia without any tibial organ. Fore tibia with one apical spur, slightly longer than maximum tibial diameter. Two spurs present on both mid and hind tibia, the posteroventral spurs about twice as long as the anteroventral ones. A distinct transverse comb of closely set posterior setulae apically on mid and hind tibia. Abdomen relatively long, bicolored, mostly dark brown, tergites 2–5 mostly yellowish, with dark dorsal triangular markings tapering proximally. Sternites 2–5 yellowish with dark apical margins. Terminalia (Figure 14D–F) dark brown, basally lighter. Tergite 9 subtriangular, slightly longer than broad. Gonocoxites fused only ventrobasally, with stronger setae along their posterior margin. Gonostylus distinctly shorter than gonocoxite, almost as long as wide, subtriangular to oval in shape, without distinct apical tooth, dorsally with a longitudinal submedial furrow.

### 3.6. Family Mycetophilidae

This is one of the largest families of Sciaroidea, comprising more than 4500 described extant species in more than 230 genera [32]. The concept and relationships among the subfamilies and particular genera vary greatly among authors. Oriental species of Mycetophilidae were studied by many authors in the past, as well as recently, e.g., References [4,5,33,34]. Two new extant species are described below.

#### 3.6.1. Genus *Hadroneura* Lundström, 1906

A small genus of Gnoristinae, with 8 species currently described from the Holarctic region. An additional new species is described below, representing the first record of the genus from Taiwan. This genus is very similar to *Dziedzickia* Johannsen, 1909, if not a subgroup of it, depending on the generic concept, which varies among authors. In any case, *Hadroneura* is an older available name, which would have priority over *Dziedzickia*. As discussed already by Ševčík et al. [4], *Dziedzickia* is a very heterogeneous genus, including several distinct species groups and many undescribed species worldwide, and, thus, needs detailed revision.

**Figure 16:**
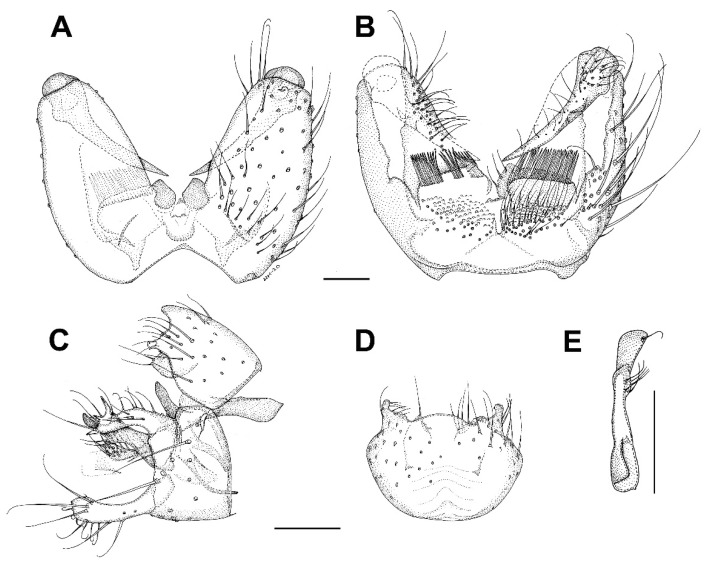
Male terminalia of Mycetophilidae and Sciaroidea *incertae sedis*. *Hadroneura martini* sp. nov. (holotype): (**A**) ventral view, (**B**) dorsal view; *Nepaletricha sikorai* sp. nov. (holotype), (**C**) lateral view, (**D**) ventral view, (**E**) gonostylus in ventral view. Scale bars: 0.1 mm.

***Hadroneura martini* sp. nov.** (Figure 1B, Figure 15B, and Figure 16A,B).

LSID urn:lsid:zoobank.org:act:A99FCE90-885E-46C2-B1DA-37F602D95D2A.

**Type material.** Holotype: male, Taiwan (China), Renai, 25.iv.2019, at light, J. Ševčík leg. (coll. NMNS).

**DNA sequences.** DNA sequence (COI barcode region) taken from the holotype (No. JSTW25) is deposited in GenBank. Its Accession number is provided in Table S1.

**Etymology.** This species is named after Dr. Martin Fikáček, a coleopterist from the National Museum in Prague. He participated at the field trip to Renai (Taiwan) where this new species was collected.

**Diagnosis.** Among the species of *Hadroneura*, the new species is quite distinctive due to its yellow flagellum and head without a proboscis.

**Description.** Male. Wing length 3.8 mm. Body mostly dark brown, except for the yellow antennal flagellum and partly yellow legs (Figure 15B). Two ocelli, near the eye margin, but not touching it. Distinct frontal suture, reaching from the antennae to the occiput. Antenna with 14 yellow cylindrical flagellomeres. Scape and pedicel yellowish brown. Mouthparts normal, not distinctly reduced or prolonged. Palpus brown, with four visible palpomeres, the apical one twice as long as the previous one. Scutum with acrostichal, dorsocentral and lateral setae; areas between them bare. Scutellum dark, covered with short setulae and several longer bristles. Laterotergite with long setae, mediotergite bare. Wing (Figure 1B) hyaline, light brown, unmarked, but with veins Rs and M_2+3_ darkened, membrane without macrotrichia. Costa only shortly produced beyond R_5_. Sc long, distally setose, ending in R_1_ between base of Rs and R_2+3_. R-m about as long as Rs. M_4_ long, starting close to wing base. Anal vein short and bare. Haltere yellow, with a dark knob. Coxae and femora dark brown, tibiae yellow, except for dark brown hind tibia. Tibiae with dense, irregularly arranged, trichia. Mid and hind tibia with several anterodorsal and posterodorsal setae. Fore tibia with a distinct tibial organ. Two yellow spurs on mid and hind tibia, front tibial spur brown. Abdomen all dark brown. Terminalia (Figure 16A,B). Tergite 9 short, transverse, densely covered with long setae, cercus about as long as tergite 9, with a subapical row of long setae. Gonostylus narrow, tapering, shorter than gonocoxite.

#### 3.6.2. Genus *Paratinia* Mik, 1874

= *Acomoptera* Vockeroth, 1980 syn. nov.

= *Drepanocercus* Vockeroth, 1980 syn. nov.

= *Loicia* Vockeroth, 1980 syn. nov.

We take the opportunity here to synonymize the genera *Acomoptera*, *Drepanocercus* and *Loicia* with *Paratinia*, the oldest available generic name in this group. Their close relationship and similarity are evident from the studies based on both molecular and morphological characters (e.g., References [32,35,36,37]). Above other characters, these four former genera share principally the same wing venation and general outline of the male terminalia. The wing venation of *Loicia* is unique due to basal branching of CuA [37], but intraspecific variation of some wing characters was demonstrated within this group, e.g., in *Drepanocercus*; see Reference [38].

Although several species groups (see Kerr [35]) may be recognized within such a broadly defined genus, they do not correspond to the genera as were defined up to the present. Species of *Loicia* and *Paratinia* share a distinct character state, setosity of the wing membrane (macrotrichia). However, this character state is known to be parallelly developed or absent even within a single genus of Sciaroidea, e.g., in *Macrocera* (Keroplatidae) or *Trichosia* (Sciaridae). Unfortunately, the setosity of wing membrane has been widely used as one of the key characters to separate the mycetophilid subfamilies Gnoristinae and Sciophilinae, leading to incorrect assignment of *Paratinia* to Sciophilinae in the past. The recent molecular study by Kaspřák et al. [32], however, clearly demonstrated that *Paratinia*, including *Acomoptera* and *Loicia*, represent a well-supported monophyletic group within Gnoristinae.

**Figure 17:**
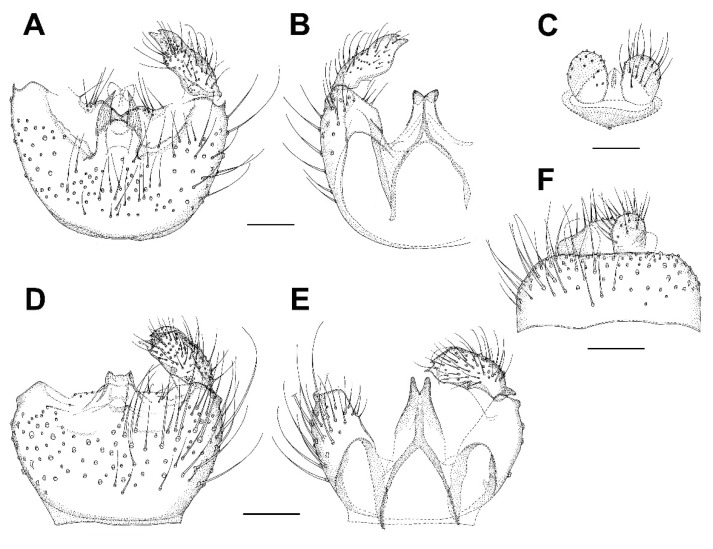
Male terminalia of *Paratinia* Mik. *Paratinia furcata* sp. nov. (holotype): (**A**) ventral view, (**B**) dorsal view; (**C**) cerci; *P. sciarina* Mik (Slovakia), (**D**) ventral view, (**E**) dorsal view, (**F**) tergite 9 and cerci. Scale bars: 0.1 mm.

***Paratinia furcata* sp. nov.** (Figure 17A–C).

LSID urn:lsid:zoobank.org:act:B9A356DE-C871-4D83-901B-3B6A1E75B3B3.

**Type material.** Holotype: male, Slovakia, Cerová vrchovina Protected Landscape Area, Gemerské Dechtáre env., Steblová skala Nature Reserve, 280 m, 27.ix.–1.xi.2017, Malaise trap, J. Roháček & J. Ševčík leg. (SMOC, specimen after DNA extraction, mounted on slide in Euparal). Paratypes (2 males): male, Czech Republic, Podyjí National Park, Havraníky, 26.iii–16.v.2002, M. Barták & Š. Kubík leg. (Malaise trap); male, the same data, except for 29.viii–28.ix. 2001 (in ethanol, both in JSL-UOC).

**DNA sequences.** DNA sequence (COI barcode region) taken from the holotype (No. JS PAR-SKB) is deposited in GenBank. Its Accession number is provided in Table S1.

**Etymology.** The specific name refers to the distinct, fork-like structure of the medioventral process of the gonocoxites (hypandrial lobe).

**Diagnosis.** This new species clearly differs from *Paratinia sciarina* Mik, 1874 in the shape of gonostylus and medioventral process of gonocoxites (see Figure 17).

**Description.** Male. Wing length 5.4 mm (holotype). Entire body dark brown. Three ocelli, lateral ones almost touching the eye margin. Antenna dark brown, with 14 elongated flagellomeres. Palpus brown, with four visible palpomeres, the apical one twice as long as the previous one. Scutum with acrostichal, dorsocentral and lateral setae; areas between them bare. Scutellum dark, with several subapical bristles. Mediotergite and all lateral thoracic sclerites bare. Wing hyaline, unmarked, membrane with sparse macrotrichia only in distal half of the wing. Costa produced beyond R5 to about 1/3 of the distance between the tips of R5 and M1. Sc long, ending in C beyond the base of Rs. R-m almost twice as long as Rs. M4 joining Cu at the level of the base of r-m. Anal vein short, but strong and setose. Haltere yellowish brown. Legs yellowish brown, tarsi darker. Tibiae densely covered with short, irregularly arranged, trichia. Fore tibia without a tibial organ. First tarsomere in all legs prolonged, about as long as tibia. Tibial spurs yellow, 1:2:2. Abdomen all dark brown. Terminalia (Figure 17A–C). Tergite 9 very short, about twice as broad as long, cerci oval, slightly longer than tergite 9, covered with setae. Gonocoxites fused, with a large medioventral sclerotized process, which is deeply forked, with branches apically pointed. Gonostylus twice as long as broad, shorter than gonocoxite, with two apical weakly sclerotized teeth.

**Discussion.** There are several undescribed species of *Paratinia*, similar to *P. furcata* sp. nov., known from various European countries (e.g., Figure 25B in Søli [36]), pending a detailed revision of this group, which is beyond the scope of this paper. In any case, a careful comparison of the male terminalia, together with DNA sequence data, is necessary to clarify the identity of all the species in this genus. There are also several undescribed Oriental species of *Paratinia* known to us (from Brunei, Taiwan, Thailand), but they will be described in a separate paper.

***Paratinia sciarina* Mik, 1874** (Figure 17D–F).

**Material examined.** Holotype: male, Austria, Freistadt, 28.vi.1868, Mik leg. (NMW).

Czech Republic: 3 males, 2 females Rejvíz, 28.ix.– 7.xi.2005, peat-bog, Malaise trap, J. Roháček & J. Ševčík leg., 4 males, Velká Kotlina Glacial Cirque, 25.vii.–4.ix.2006, Malaise trap, J. Roháček & J. Ševčík leg.; Slovakia: male, Červená Skala env., Trsteník, 16.vi.– 7.vii.2016, Malaise trap, J. Roháček & J. Ševčík leg. (specimen figured, on slide in Euparal, after DNA extraction, No. JSS6).

**DNA sequences.** DNA sequence (COI barcode region) taken from the specimen from Slovakia (published by Kaspřák et al. [32]) is deposited in GenBank. Its Accession number is in Table S1.

**Discussion.** The type series of this species is deposited in the Naturhistorische Museum Wien (Austria) and includes 4 males, out of which one is labeled as “Type”. The Czech and Slovak specimens were compared with the holotype to confirm their identity. Females of this species are reported here for the first time. They have white apical segments of antennae, similar to the Nearctic *Paratinia recurva* Johannsen, 1910 (see Taber [39]).

### 3.7. Sciaroidea Incertae Sedis

This is a group of some 20 genera of Sciaroidea, which have not yet been definitely assigned to a family. These taxa were traditionally treated as the *Heterotricha* Loew, 1850, group but, in recent years, have been referred to as Sciaroidea incertae sedis [2,40,41,42]. Two new species from this enigmatic group are described below, one of them in a new fossil genus.

***Burmatricha* gen. nov.**

LSID urn:lsid:zoobank.org:act:8C2C57FD-C392-4B6A-A1AE-4B064E8A8112.

**Type species.** *Burmatricha mesozoica* sp. nov.

**Gender.** Feminine.

**Etymology.** The generic name is derived from „Burma“ (= Myanmar), the country of the origin of the amber, and “*Heterotricha*”, a similar genus of Sciaroidea.

**Diagnosis.** Wing length approximately 1.6–1.8 mm. Antennae as long as wing, with 14 prolonged flagellomeres. Wing venation similar to that of *Heterotricha* Loew, 1848 and related genera. Macrotrichia on wing membrane absent. Sc relatively short, ending in C. Costa long, reaching almost to M_1_. Vein R_2+3_ absent. Veins r-m, m-m and m-cu in one line. about half as long as R_5_, r-m present or absent. Stem of M-fork long. CuA strong and downcurved. Both CuP and anal veins not traceable. Male terminalia with simple gonostyli, as long as gonocoxites, narrow, strongly sclerotized in the posterior half and apically rounded. For more information, see the description of the type species below.

***Burmatricha mesozoica* sp. nov.** (Figure 9D and Figure 10B,C).

LSID urn:lsid:zoobank.org:act:DF085879-B80A-4EBF-B944-C26C0D5B5A14.

**Type material.** Holotype: male, No. 118/2020, Burmese amber (the earliest Cenomanian, 98.79 ± 0.62 Ma), deposited in the Silesian Museum, Opava, Czech Republic (SMOC).

Paratypes (6 males, all Burmese amber inclusions): male No. 17/2020 (coll. SMOC), male No. 54/2020 (NMPC), male No. MP/4343 (coll. ISEA PAS), male No. 148/2021 (coll. JSL-OUC); two males (No. 102/2021a and 102/2021b) in one piece of Burmese amber (syninclusion No. 102/2021, coll. JSL-OUC).

**Diagnosis.** This species is recognizable from the other members of the *Heterotricha* group (=Sciaroidea *incertae sedis*) by the short Sc, ending free, long C, the absence of macrotrichia on wing membrane, veins r-m, m-m and m-cu in one line, and the simple structure of male terminalia.

**Description.** Male. Wing length approximately 1.7 mm (holotype). Head with relatively large compound eyes, which are emarginated near antennal base. Palpi with three visible segments. Antennae relatively long, as long as the wing, with 14 flagellomeres, F5–F14 about 8 times as long as broad. Wing relatively narrow, with ratio of length to width about 3.1. Macrotrichia on wing membrane absent. Sc ending in C before the level of Rs. C distinctly produced, reaching almost to the apex of M_1_. Vein R_2+3_ absent. Veins r-m, m-m and m-cu in one nearly horizontal line. Stem of M-fork long, slightly longer than M_1_. CuA strong and downcurved. Both CuP and anal veins not traceable. Legs covered with trichia and several sparse posterior setae on mid and hind tibia. Tibial spurs 1:2:2. Terminalia (Figure 10B) with tergite 9 short, about twice as broad as long. Cerci short but longer than T9. Gonostylus simple, unbranched, as long as gonocoxites, narrow, strongly sclerotized in the posterior half and apically rounded.

**Discussion.** The phylogenetic position and interrelationships of various genera of the Sciaroidea *incertae sedis* are a real puzzle, which is beyond the scope of this paper, but preliminary molecular data suggest that these genera do not constitute a monophyletic group [2,32]. The family placement of these genera is also obscure, although there are some indications that they could belong to the Mesozoic family Antefungivoridae [41].

***Nepaletricha sikorai* sp. nov.** (Figure 16C–E).

LSID urn:lsid:zoobank.org:act:CB57DD0E-3E3A-437E-99FE-C27E62266A16.

**Type material.** Holotype: male, Thailand, Chiang Mai, Ban Mae Lao, 21.v.–2.vi. 2017, M. Mantič, T. Sikora & M. Tkoč leg., Malaise trap 2 (SMOC, on slide in Euparal, specimen after DNA extraction).

**DNA sequences.** DNA sequence (COI) taken from the holotype (No. JSBA25A) is deposited in GenBank. Its Accession number is provided in Table S1.

**Etymology.** This species is named after Mr. Tomáš Sikora (University of Ostrava, Czech Republic), a student of lower gall midges, who collected the type specimen.

**Diagnosis.** *Nepaletricha sikorai* sp. nov. is similar to *N. dembickyi* Hippa and Ševčík, 2014 and *N. montana* Hippa and Ševčík, 2014. All three differ from the other *Nepaletricha* by having the gonocoxites posterolaterally divided into posteriorly directed large lobes, longer than the undivided anterior part. In *N. dembickyi* and *N. montana*, there is one more ventral and one more dorsal lobe, while, in *N. sikorai* sp. nov., there are two more dorsal ones, both equal in size. All the three species are different in many details in their terminalia (Figures 12G,H and 2A,B in Hippa and Ševčík [41], Figure 4A–D in Hippa et al. [43]).

**Description.** Male. Wing length 3.6 mm. Head. Color of head brown, antenna and maxillary palpus slightly paler beyond the middle of flagellomere 1. Face with two setae medioventrally. Maxillary palpus with palpomere 5 twice the length of palpomere 4, palpomere 2 with dome-like sensilla mesially on distal part, palpomere 3 with a patch of closely placed hyalinous sensilla ventrolaterally, palpomere 1 with 3 setae, 2 with 2 setae, 3 with 9 and 5 with 8 setae. Antenna flagellomeres 4 and 14 similar to *Nepaletricha furcata* Hippa & al. (Figure 1D,F in Reference [43]), flagellomere 14 with apical ovate appendix, flagellomere 1 6.7 and flagellomere 4 3.4 times longer than broad. Thorax. Brown. Scutum with ca. 7 lateral setae, ca. 8 dorsocentral setae and with 0 acrostichal setae, scutellum with 4 setae and prothoracic epimeron with 3–4 setae. Legs. Coxae and trochanters brown, other part of legs pale brown. Front leg with parts distal to femur lost. Coxae similar to *N. furcata* (Figure 1A in Reference [43]). Wing. Similar to *N. furcata* (Figure 2B in Reference [43]). Colour of wing and haltere brownish. Wing length 2.2 (2.5) mm.: Sc setose dorsally, R and R1 setose dorsally, R1 distally setose on both sides, R_4+5_ setose dorsally and ventrally, stM, M1 and M2 setose dorsally and ventrally, CuA1 and CuA2 setose dorsally and A1 setose dorsally; wing membrane setose dorsally and ventrally in the reminiscent way as in *N. furcata* except costal cell is nonsetose. Abdomen. Brown. Segment 8 similar to *N. dembickyi* (Figure 3C in Reference [41]), sternite nonsetose. Terminalia (Figure 16C–E) brownish, tergite 9 posterolaterally with small setose lobe. Gonocoxite deeply cut into three large posterolateral setose lobes, the two more dorsal ones with several sublobes. Gonostylus straight, with one subapical and a few submedial setae. Aedeagus and parameres not seen in detail in the mount. Cerci small, elongate with a few long marginal setae. Sternite 10 large, medially not divided into two halves, with a few long posterolateral setae. Female. Unknown.

## Data Availability

Not applicable.

## Supplementary Materials

The following are available online at https://www.mdpi.com/article/10.3390/insects13010019/s1, Table S1: List of specimens used for DNA extraction, with GenBank accession numbers.
