# Supplementary material for: Just a Fragment of Undescribed Diversity: Twenty New Oriental and Palearctic Species of Sciaroidea (Diptera), including DNA Sequence Data and Two New Fossil Genera
Source: Insects. 2021 Dec 23;13(1):19. doi: 10.3390/insects13010019 (PMC8779109; doi:10.3390/insects13010019)
Supplement: Supplementary file 1 [file insects-13-00019-s001.zip › insects-1504552-supplementary.pdf]

Table S1. List of specimens used for DNA extraction, with GenBank accession numbers

Species	Voucher code	Sampling locality and year	COI	28S	16S
Asioditomyia bruneicola **sp. nov.**	JSD5	Brunei, 2014	KT316838	KP288793	-
	JSD5B	Brunei, 2014	OL743128	-	-
Asioditomyia japonica (Sasakawa, 1963)	JSD10	Japan, 2015	OL743144	OL741620	-
Asioditomyia lacii **sp. nov.**	JSTW38	Taiwan, 2018	OL743120	OL741621	-
Bibio marci (Linnaeus, 1758)	JSOUT1	Czech Republic, 2013	KT316846	KJ136761.1	-
Bolitophila nikolae Ševčík **sp. nov.**	JSBN20	Taiwan, 2019	OL743127	-	-
Catocha angulata Jaschhof, 2009	JSC26	Slovakia, 2014	KT316837	-	KP288711
Catocha brachycornis (Spungis & Jaschhof, 2000)	JSC86	Estonia, 2015	MG684831	-	N/A
Catocha incisa Jaschhof, 2009	JSC84	Czech Republic, 2016	MG684811	-	MG684522
Catocha jingfui **sp. nov.**	JSTW32B	Taiwan, 2019	OL743123	-	OL711947
	JSTW32D	Taiwan, 2019	OL743140		
Catocha latipes Haliday, 1833	JSBA51	Czech Republic, 2020	OL743121	-	OL711945
Catocha manmiaoe **sp. nov.**	JSTW32I	Taiwan, 2019	OL743124	-	OL711948
	JSTW32L	Taiwan, 2019	OL743137	-	-
Catocha shengfengi **sp. nov.**	JSTW32	Taiwan, 2019	OL743122	-	OL711946
	JSTW32C	Taiwan, 2019	OL743136	-	-
Catotricha subobsoleta (Alexander, 1924)	JSOUT42	USA, 2014	KT316873	-	MG554124
Celebesomyia inocellata Saigusa, 1973	JSD8	Indonesia, 2007	OL743119	OL741618	-
Chetoneura davidi **sp. nov.**	JSK59	Brunei, 2015	MT446924	-	-
	JSK59B	Brunei, 2014	OL743129	-	-
Diadocidia pseudospinusola **sp. nov.**	JSTW28B	Taiwan, 2018	OL743134	-	-
	JSTW28	Taiwan, 2019	OL743133	-	-
Ditomyia asiatica **sp. nov.**	JSBA46	Thailand, 2014	OL743118	OL741615	-
	JSBA46D	Thailand, 2014	OL743126	-	-
Ditomyia fasciata (Meigen, 1818)	JSSJ3	Czech Republic, 2010	-	KJ136770	-
	JSDITFAS	Czech Republic, 2013	MT446886	-	-
Ditomyia macroptera Winnertz, 1852	JSD1A	Slovakia, 2013	OL743141	OL741616	-
Euceroplatus mantici **sp. nov.**	JSBA24	Thailand, 2017	MT446879	-	-
Hadroneura martini **sp. nov.**	JSTW25	Taiwan, 2019	OL743139	-	-
Lestremia cinerea Macquart, 1826	JSOUT6	Czech Republic, 2014	KJ136765	-	KJ136728
Nepaletricha sikorai **sp. nov.**	JSBA25A	Thailand, 2017	MT446880	-	-

Nervijuncta sp.	JSD11	New Zealand, 2016	MT446884	MT446784	-
Paratinia furcata **sp. nov.**	JSPAR-SKB	Slovakia, 2017	OL743130	-	-
Paratinia sciarina Mik, 1874	JSS6	Slovakia, 2016	KC435658	-	-
Penthetria funebris Meigen, 1804	JSOUT14	Slovakia, 2014	KT316858	KP288804	-
Planetella taiwanensis **sp. nov.**	JSPLA5	Taiwan, 2016	OL743131	-	-
Platyceridion yunfui **sp. nov.**	JSK81A	China, 2016	MT446937	-	-
Rhipidita sp.	JSD9	French Guiana, 2012	OL743143	OL741619	-
Setostylus fangshuoi **sp. nov.**	JSTW36	Taiwan, 2019	MT446958	-	-
	JSTW36B	Taiwan, 2019	OL743138	-	-
Symmerus annulatus (Meigen, 1830)	JSD2	Slovakia, 2012	KX453757	KX453708	-
Symmerus kubani Sevcik, 2000	JSD4	Thailand, 2007	OL743142	OL741617	-
Symmerus nobilis Lackschewitz, 1937	JSB3	Slovakia, 2012	KT316831	KJ136751	-
Symmerus pectinatus Saigusa, 1966	JSTW46	Taiwan, 2019	OL743125	OL741622	-
Terocelion adami **sp. nov.**	JSTW31	Taiwan, 2019	OL743135	-	-
